# Round Robin for Optical Fiber Bragg Grating Metrology

**DOI:** 10.6028/jres.105.066

**Published:** 2000-12-01

**Authors:** A. H. Rose, C.-M. Wang, S. D. Dyer

**Affiliations:** National Institute of Standards and Technology, Boulder, CO 80303

**Keywords:** dispersion, metrology, optical fiber Bragg grating, optical fiber sensors, reflectance, relative group delay, transmittance, wavelength division multiplexing

## Abstract

NIST has administered the first round robin of measurements for optical fiber Bragg gratings. We compared the measurement of center wavelength, bandwidth, isolation, minimum relative transmittance, and relative group delay among several grating types in two industry groups, telecommunications and sensors. We found that the state of fiber Bragg grating metrology needs improvement in most areas. Specifically, when tunable lasers are used a filter is needed to remove broadband emissions from the laser. The linear slope of relative group delay measurements is sensitive to drift and systematic bias in the rf-modulation technique. The center wavelength measurement had a range of about 27 pm in the sensors group and is not adequate to support long-term structural monitoring applications.

## 1. Introduction

At the request of industry, NIST has administered the first round robin of measurements for optical fiber Bragg gratings (FBG). In this paper we report on that work. Fiber Bragg gratings are wavelength-selective reflectors that can be written into the core of optical fibers. These relatively new components are extremely important for telecommunication and sensor applications. In new wavelength division multiplexed (WDM) optical fiber communication systems FBGs are used as wavelength filters and dispersion compensators (dispersion in optical fiber spreads optical pulses in time and limits the data rate of the fiber link.). Also, FBGs make excellent strain sensors that can be networked to obtain distributed strain measurements of large structures, such as bridges and ships. In spite of the numerous and growing commercial applications of fiber Bragg gratings, there are no standard measurement procedures for the gratings and a variety of definitions are being used for important measurement parameters.

At an informal meeting during the Optical Fiber Communication Conference in February of 1999 (OFC’99), a quorum of industry representatives recommended measuring several specific spectral and relative group delay (*RGD*) properties of a chirped FBG. (The derivative of *RGD* versus wavelength gives a measure of dispersion in an optical component.) In June of 1999 NIST launched the round robin among two industry groups; one group named “Telecom” measured spectral properties and *RGD* for their gratings, the other industry group named “Sensors” measured only the spectral properties of their gratings. The round-robin participants in the two groups were: ADC, Agilent, Corning, Perkin Elmer, GNnet-test, NPL, and 3M in the telecom group, and Blue Road Research, CiDRA, EXFO, Micron Optics, and NRL in the sensors group. Raw data from the participants were sent to NIST because no formal methods for analyzing the spectral or *RGD* data existed. The participants were each sent a set of instructions to follow, which are presented in Appendices A and B.

The purpose of this round robin was to determine the state of industrial metrology concerning spectral and *RGD* measurements in FBGs. From the data and results collected we are making recommendations on the methods of measurement and analysis for FBG.

**Fig 1:**
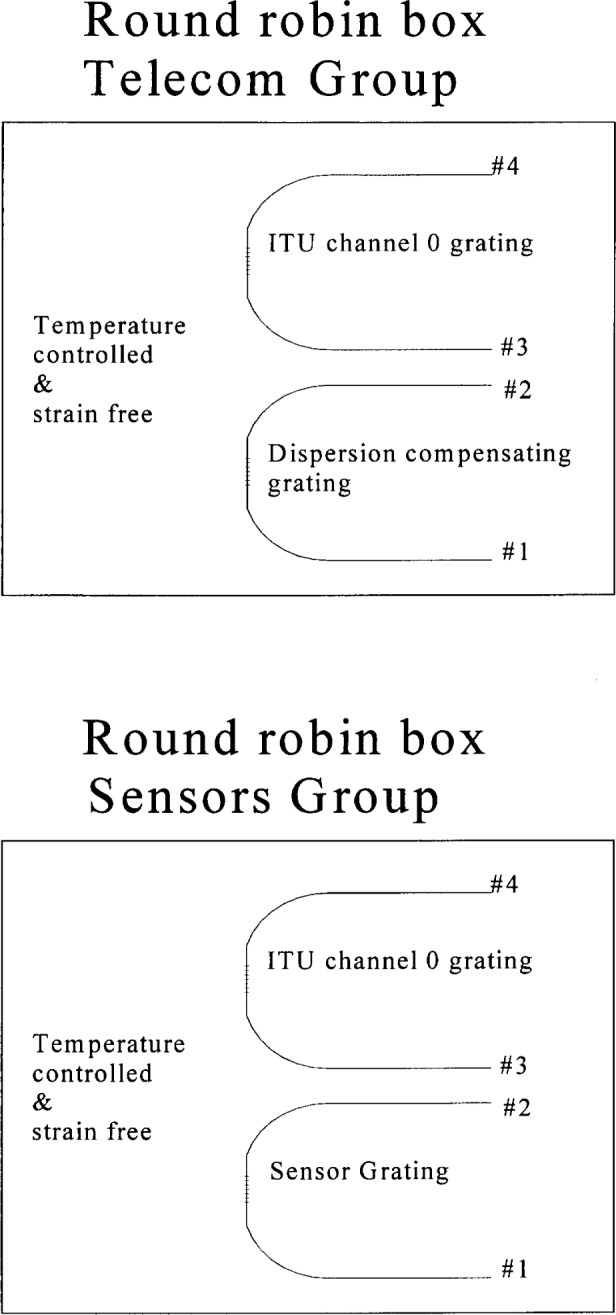
Round robin box configurations.

The participants each received a box containing two gratings with about 10 m of Corning SMF-28 fiber^1^ spliced on each side of the grating. Figure 1 shows a sketch of the box and gratings. The telecom group received an International Telecommunication Union (ITU) channel 0, 50 GHz bandwidth grating and a mildly chirped, ≈ 16 nm bandwidth, grating. The sensor group received a similar ITU channel 0, 50 GHz bandwidth grating and a narrow-bandwidth, low reflectance, sensor grating. To stabilize the ITU and chirped gratings against temperature changes they were packaged in an athermal package and placed on a thermoelectric cooler (TEC), controlled to within less than 0.1 °C. The sensor grating was packaged in a silicon rubber tube, strain free, and placed on the same TEC. Again, the TEC was controlled to within less than 0.1 °C.

The raw data from the each of the participants are shown in Appendices C and D. The labels A, B, C, etc. on the raw data and analysis represent participants and were determined by a random number generator.

During the OFC’99 meeting the participants agreed that NIST should determine an analysis method and apply it to the raw data to determine a grating parameter of interest. If formal methods were determined at a later time, the data could be re-analyzed with those methods and valuable insight could be gained without having to repeat the round robin.

For transmittance measurements, most of the participants launched on port No. 1 or No. 4, of Fig. 1. Reflectance data were obtained with the aid of a coupler (participant supplied) and only one port; usually No. 1 or No. 4 in Fig. 1 was used. Relative values for transmittance and reflectance were used since only relative features were needed to determine many of the parameters.

## 2. Measurement Techniques

Measurement techniques used by the various participants depended on the group. The telecom group used primarily a grating-tuned diode laser, power meter, and wavelength meter system for the spectral measurements. The sensors group used primarily a wide band source and an optical spectrum analyzer for spectral measurements. In some cases a swept diode laser or wideband source and swept fiber Fabry-Perot filter were used.

**Fig. 2:**
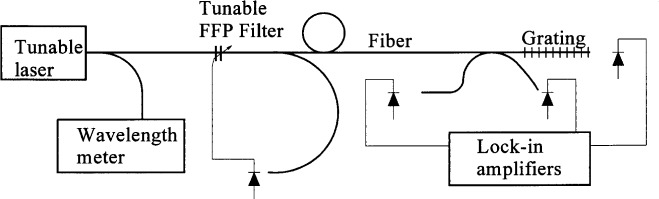
NIST spectral measurement system.

The NIST system employed for spectral measurements is shown in Fig. 2. A tunable fiber Fabry-Perot filter (FFP) was used to filter amplified spontaneous emission-light (ASE) from a grating-tuned diode laser. The ASE was filtered, because the measurement system photodetectors respond to light over a wide bandwidth. The ASE power, though small over narrow bandwidths, becomes significant over the photodetector bandwidth. ASE represents spectral noise on the narrow spectral laser output and will limit the dynamic range of the measurement system. A wavelength meter provided the wavelength scale. Uncertainty analysis for this system is presented in Appendix E.

**Fig. 3:**
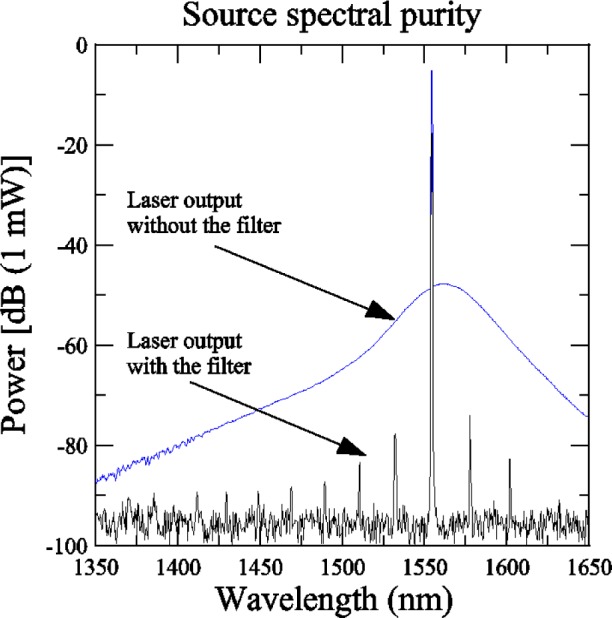
Amplified spontaneous emission from a tunable laser diode with and without a tunable Fabry-Perot filter.

**Fig. 4:**
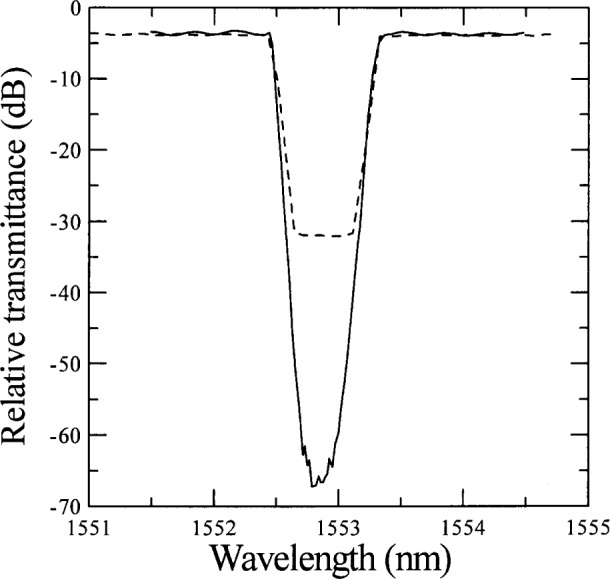
The effect of amplified spontaneous emission-light (ASE) on the measurement of transmittance in a fiber Bragg grating. The dashed line represents data taken with out any ASE filtering and the solid line represents data taken with a 1.52 GHz tunable Fabry-Perot filter.

The effect of the FFP filter can be seen in Figs. 3 and 4. Figure 3 shows the unfiltered and filtered output of the tunable laser over the 1350 nm to 1650 nm band. These data were taken with a 1 nm resolution, optical spectrum analyzer. The laser peak power at 1553 nm was about 308 μW and the integrated ASE across the measured spectra in Fig. 3 was about 1.7 μW. The ratio of these powers is about 23 dB and is a good measure of the dynamic range of the system without the FFP. With the FFP filter the ASE is suppressed, as shown in Fig. 3. With the FFP filter the laser power is at about 174 μW, and the integrated ASE power is about 76 pW for a power ratio of about 64 dB. If a second FFP filter with a different free spectral range was used, further ASE suppression could be realized.

Figure 4 shows the effect of the ASE on a measurement of a FBG’s relative transmittance. Without the FFP filter the minimum relative transmittance is only about 25 dB. With the FFP filter the minimum relative transmittance is about 65 dB. Laser ASE will also affect the isolation or crosstalk measurement of a FBG.

**Fig. 5:**
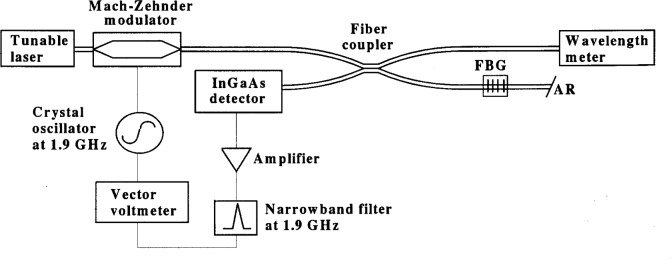
NIST phase-shift measurement system for relative group delay. The fiber after the fiber Bragg grating (FBG) has an anti-reflection (AR) termination.

The *RGD* of the chirped grating was determined by various phase-shift techniques. The NIST phase-shift system is shown in Fig. 5. A detailed description can be found in Ref. [1] and a summary of the uncertainty is presented in Appendix E.

**Fig. 6:**
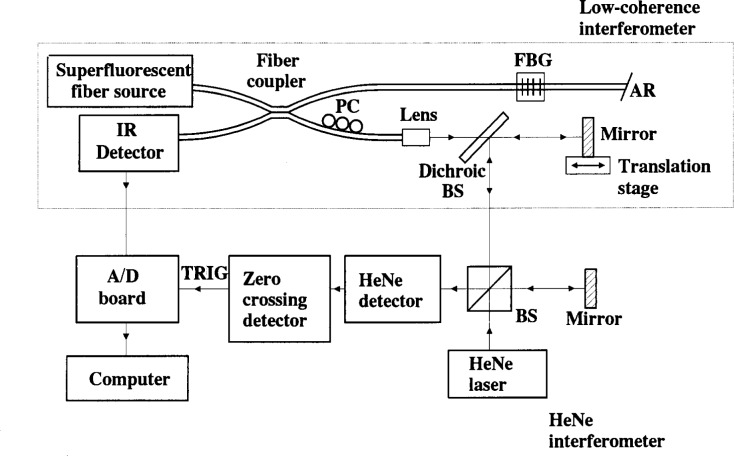
NIST low-coherence interferometer for relative group delay measurements. For maximum fringe visibility the low-coherence interferometer has a fiber polarization controller (PC).

NIST also employed a new, low-coherence interferometer method to determine the *RGD* of the chirped grating. That system is shown in Fig. 6 and details of this system are described in the literature [2, 3]. A full uncertainty analysis is in progress.

## 3. Analysis Methods

At the time of this report, a fiber-optic test procedure (FOTP) titled, “Amplitude response measurement of narrow-band, passive fiber optic components,” was being written by the Telecommunication Industry Association (TIA) FO-6.3.5 Subcommittee on Fiber Optic Interconnecting Devices. NIST is working with this subcommittee on the FOTP and any appropriate results from this round robin will be included in the FOTP. Another FOTP on dispersion measurements is also being drafted by the same subcommittee.

From the TIA sources and the meeting at OFC’99, several parameters of interest were identified that could be obtained from the raw data files sent to NIST. The spectral parameters of interest to TIA and the OFC’99 group were center wavelength, bandwidth, isolation, and transmittance. The *RGD* parameters of interest were the linear slope across the operating bandwidth of the grating and the ripple magnitude and period.

To determine the center wavelength, bandwidth, and isolation of a grating from the reflectance data, NIST used the following methods (see also Appendices F and G). First, the maximum reflectance in the plateau region was determined. Then, wavelengths at reflectance values of −3 dB and −0.5 dB from the maximum plateau reflectance were found by interpolating between data. The center wavelength λ_c_ is defined as (λ_+_ − λ_−_)/2, and the bandwidth is defined as (λ_+_ − λ_−_), where λ_+_ and λ_−_ are the wavelengths at data values −*x* dB (*x* = 0.5 or 3) on each side of the plateau region.

A similar approach was used to find the isolation *I* of a grating. The isolation was determined by the maximum reflectance value *M* and the maximum out-of-band reflectance value *O* in an adjacent channel (*I* = *M − O*). The ITU channel center frequency spacing is defined as (193.1 + *J × CS*) THz where *J* is an integer and *CS* is the channel spacing [4]. The integer *J* is either even or odd. An adjacent channel would be a channel where *J*_a_ = *J ± n* and *n* is an integer.

The minimum relative transmittance was determined from relative transmittance data by fitting a spline function to the data and locating the minimum.

From the *RGD* data, the linear slope of the chirped grating was determined from data within the −3 dB reflectance bandwidth (determined from a robust method described in Appendix F) using a least-squares fit. The residual *RGD* is found by subtracting the linear slope from the *RGD* reflectance data. The ripple magnitude and period varied across the bandwidth of the chirped grating. We constructed two simple methods for comparing the *RGD* ripple between the different participants.

## 4. Telecom Group

### 4.1 Grating History

To assure that there was no bias to a grating measurement as the round robin progressed, periodically we returned the round robin boxes to NIST for measurement. In this section a history of the center wavelength of each of the telecom gratings and the linear slope of the *RGD* of the chirped grating is given as a function of time.

At the end of the first 60 days of the telecom round robin, the box received a shock that caused a splice tray to come lose from its moorings and break some fiber leads inside the box. After repairs were made at NIST, the gratings in the telecom box showed a change in the center wavelength that exceeded the uncertainty of our system. Data from participants that subsequently measured the telecom gratings received an appropriate wavelength correction.

**Fig. 7:**
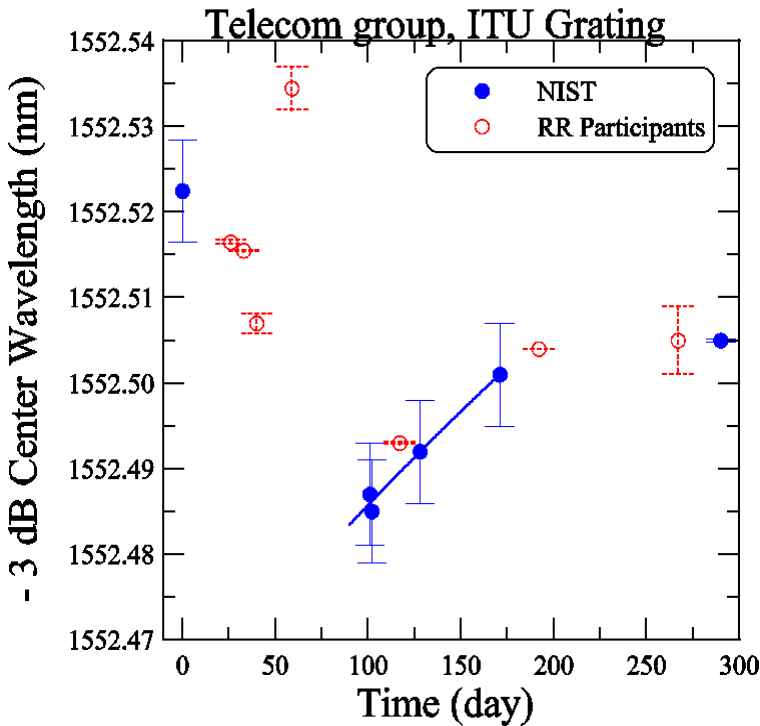
History of the telecom group ITU grating center wavelength. NIST uncertainties are expanded uncertainties, coverage factor (*k* = 2) and include all known components of uncertainty. Participants’ uncertainties are the fit uncertainty only (two standard deviation estimate).

Figure 7 shows the center wavelength versus time for the telecom ITU grating. The NIST measurements are solid dots and those from the round-robin participants are open circles. The uncertainties on the NIST data represent the expanded uncertainty of our measurement system, *U* = 6 pm (two standard deviation estimate and hence a coverage factor of *k* = 2, taking into account all known components of uncertainty). This uncertainty was subsequently reduced to *U* = 0.2 pm. The uncertainties on the round robin participants’ measurements represent only the fit uncertainty (two standard deviation estimate) and are shown as a dashed line. The center wavelength is determined from the mean of the −3 dB wavelength values. After the box was returned due to damage, a shift in the center wavelength of about 37 pm was recorded and the grating center wavelength was monitored periodically afterwards. To give a fair correction to the later round robin participants, a linear least-squares-fit was applied to the NIST measurements that just preceded and followed a participant or group of participants. The fit is shown as the solid line in Fig. 7. A correction factor was obtained that gives a center wavelength equal to the beginning NIST value.

**Fig. 8:**
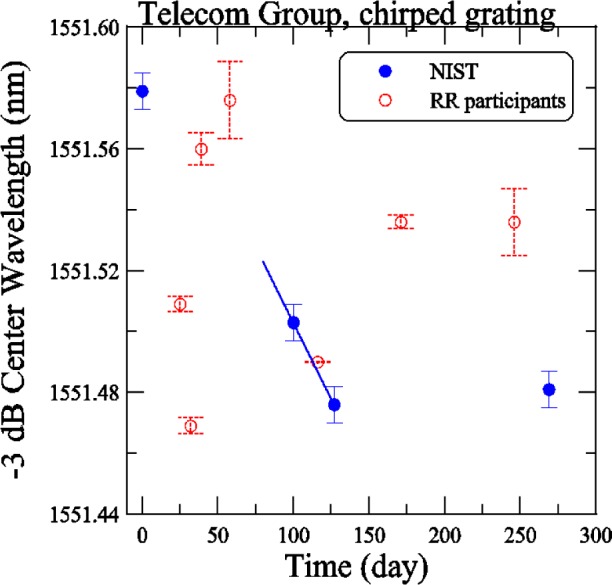
History of the Telecom Group’s center wavelength of the chirped grating. NIST uncertainties are expanded uncertainties, coverage factor (*k* = 2) and include all known components of uncertainty. Participants’ uncertainties are the fit uncertainty only (two standard deviation estimate).

Figure 8 shows the −3 dB center wavelength versus time for the telecom chirped grating. The NIST measurements are solid dots and the round robin participants are open circles. The uncertainties shown were determined in the same way as in Fig. 7. A shift in the center wavelength of 75 pm was recorded after the box was repaired. A linear least-squares-fit to the NIST measurements is shown as a solid line in Fig. 8 and a correction factor was determined for the comparison of subsequent participants.

**Fig. 9:**
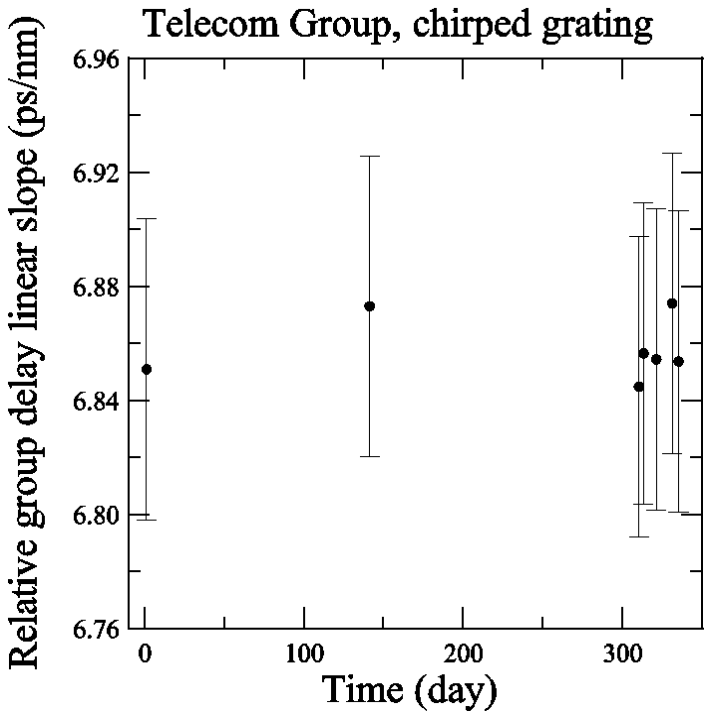
History of the linear slope of the relative group delay (*RGD*) of the Telecom Group’s chirped grating.

Figure 9 shows the *RGD* linear slope history of the telecom chirped grating versus time in days. The history was obtained from the low-coherence interferometer system and shows no appreciable change over 350 days. The uncertainties shown represent the repeatability of this system, 0.053 ps/nm (two standard deviation estimate; full uncertainty analysis is being conducted). Apparently the damage done to the system did not affect the *RGD* linear slope and no correction was added to the participants values.

These histories show that the gratings remained stable enough for the telecom round robin to be useful. When they did drift, periodic measurements at NIST corrected the bias.

### 4.2 Summary of Telecom Group Results

From the data received from each participant, the following parameters were determined: the center wavelength and bandwidth were found using the −3 dB and −0.5 dB points from the relative reflectance of the ITU grating. Also, the isolation was determined for the ITU grating. From the relative transmittance data, a minimum relative transmittance was measured. From the relative reflectance of the chirped grating, the center wavelength and bandwidth were found at −3 dB, and the minimum relative transmittance was determined from the relative transmittance data. From the *RGD* data, the linear slope was determined over the −3 dB bandwidth (see Appendix F) by a least-squares-fit method. Also, a comparison of the *RGD* ripple among the participants was made.

#### 4.2.1 ITU Grating

Several comments can be made about the relative reflectance data for the ITU grating (Appendix C, Fig. 29). Participants C, E, and F all had coarse data sets (large wavelength intervals between values), so that not enough data were obtained in the plateau region and band edges for reliable measurements to be made. In the following figures, these coarse data sets increase the fit uncertainty and bias the center wavelength, bandwidth, and isolation measurements. Participant D may not have normalized the reflectance to source power fluctuations, as the ITU grating reflectance in the plateau region has more noise than any other participant; see Fig. 29. The −0.5 dB bandwidth is very sensitive to the shape of the ITU grating reflectance.

**Fig. 10:**
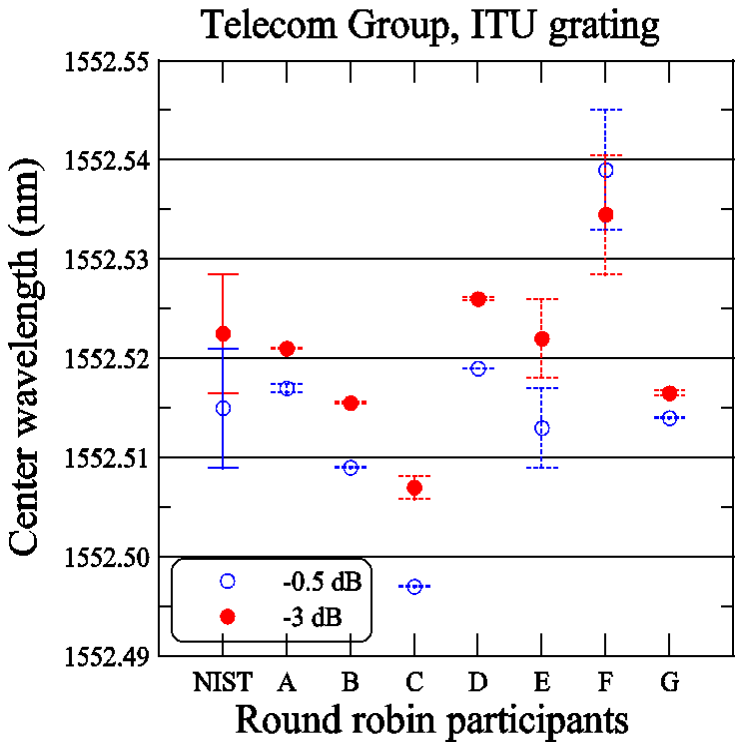
Center wavelength of the Telecom Group’s ITU grating. NIST uncertainties are expanded uncertainties, coverage factor (*k* = 2) and include all known components of uncertainty. Participants’ uncertainties are the fit uncertainty only (two standard deviation estimate).

Figure 10 shows the results of the ITU center wavelength determined using the −3 dB and −0.5 dB points. The expanded uncertainties for the NIST measurement are 6 pm (*k* = 2) and take into account all known components of uncertainty. The uncertainties for the participants are just the fit uncertainty (two standard deviation estimate). For the −3 dB center wavelength, the range of values is 27.5 pm, the standard deviation *s* is 8 pm, and the mean is 1552.521 nm. For the −0.5 dB center wavelength, the range of values is 42 pm, the *s* = 12 pm, and the mean is 1552.515 nm.

The difference in the −3 dB and −0.5 dB center wavelength values is about 6 pm. These mean values are only −5 pm (−3 dB measurement) and −11 pm (−0.5 dB measurement) from the ITU channel 0 wavelength of 1552.526 nm. For either criterion (−0.5 or −3 dB), this grating would pass the Telcordia specification that the specified and actual center wavelengths differ by less than 20 % (10 GHz or 80 pm in this case) of the specified bandwidth [4].

**Fig. 11:**
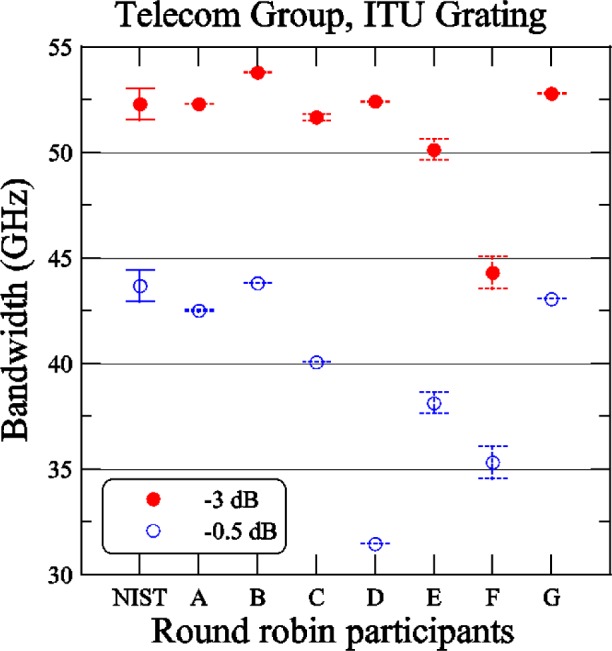
Bandwidth of the Telecom Group’s ITU grating. NIST uncertainties are expanded uncertainties, coverage factor (*k* = 2) and include all known components of uncertainty. Participants’ uncertainties are the fit uncertainty only (two standard deviation estimate).

Figure 11 shows the results of the ITU bandwidth determined at −3 dB and −0.5 dB. The uncertainties are the same as discussed for Fig. 10 converted to GHz (the NIST uncertainty is 748 MHz). For the −3 dB bandwidth the range of values is 9.5 GHz, the standard deviation *s* is 3 GHz, and the mean is 51.2 GHz. In most cases the participants would pass this as a 50 GHz ITU grating using the −3 dB criterion. For the −0.5 dB bandwidth the range of values is 12 GHz, the standard deviation *s* is 4 GHz, and the mean is 39.7 GHz. In all the cases the round-robin participants would reject this grating as a 50 GHz ITU grating at the −0.5 dB criterion. The difference in the mean bandwidth values from −3 dB to −0.5 dB is −11.5 GHz.

**Fig. 12:**
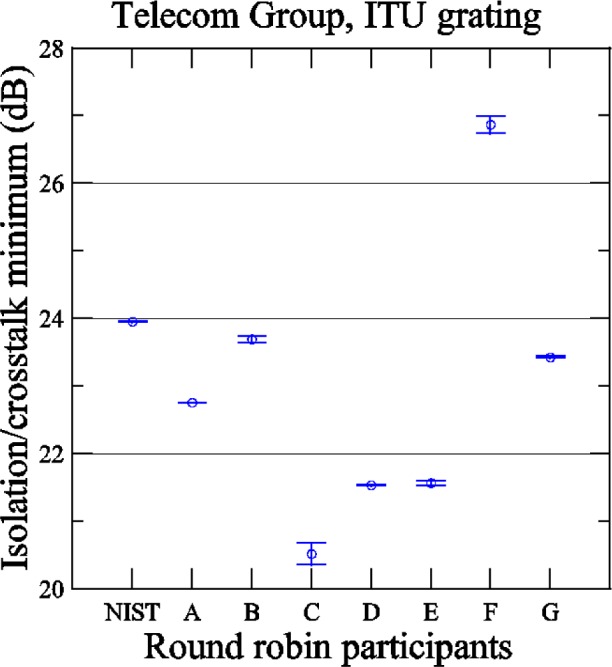
The isolation for the Telecom Group’s ITU grating.

Figure 12 shows the results of the isolation measurement of the ITU grating determined by finding the value of the plateau region of the reflectance data and the highest reflectance value in an adjacent channel. The uncertainties are the fit uncertainty only. Telcordia specifies that the isolation for a branching/filtering component should be at least 25 dB for data rates up to 10 Gb/s [4]. For most participants this grating would not pass this test.

**Fig. 13:**
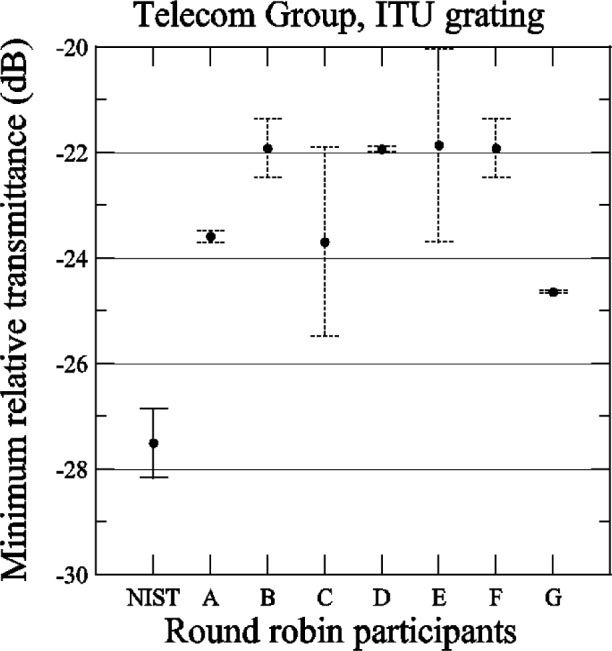
Minimum relative transmittance for the Telecom Group’s ITU grating. NIST uncertainties are expanded uncertainties, coverage factor (*k* = 2) and include all known components of uncertainty. Participants’ uncertainties are the fit uncertainty only (two standard deviation estimate).

Figure 13 shows the values for the minimum relative transmittance of the ITU grating taken from the relative transmittance data shown in Appendix C, Fig. 30. For strong gratings the minimum relative transmittance measurement is quite sensitive to the spectral purity of the laser light source. NIST’s fiber Fabry-Perot filtered laser shows the lowest minimum transmittance. The uncertainties shown are only the uncertainty of the fit (two standard deviation estimate).

#### 4.2.2 Chirped Grating

The data sets for the chirped grating are shown in Appendix C, Figs. 31, 32, and 33. These data sets were used to measure the center wavelength, bandwidth, minimum relative transmittance, and *RGD* linear slope and ripple. Participants C and F provided coarse data sets. Again, because the data interval is large, the uncertainties of the fits to their data are larger than those for the other participants, and in some cases the results are biased. Because of the coarseness of the C and F data sets, no ripple information could be determined from these data.

**Fig. 14:**
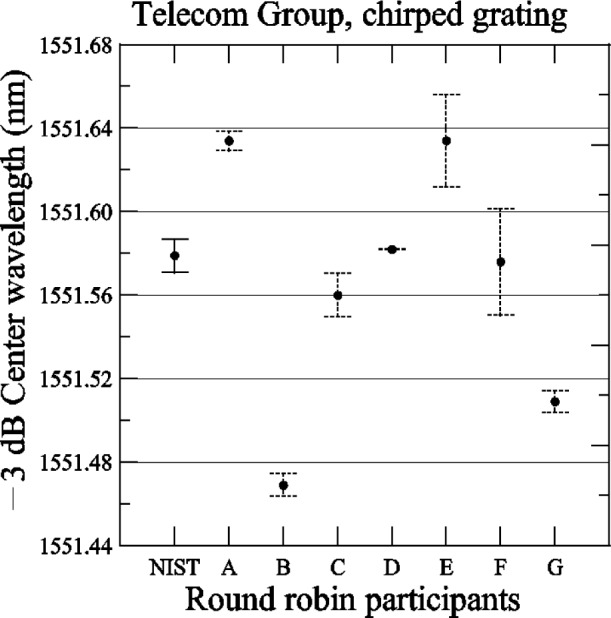
Center wavelength of the Telecom Group’s chirped grating. NIST uncertainties are expanded uncertainties, coverage factor (*k* = 2) and include all known components of uncertainty. Participants’ uncertainties are the fit uncertainty only (two standard deviation estimate).

**Fig. 15:**
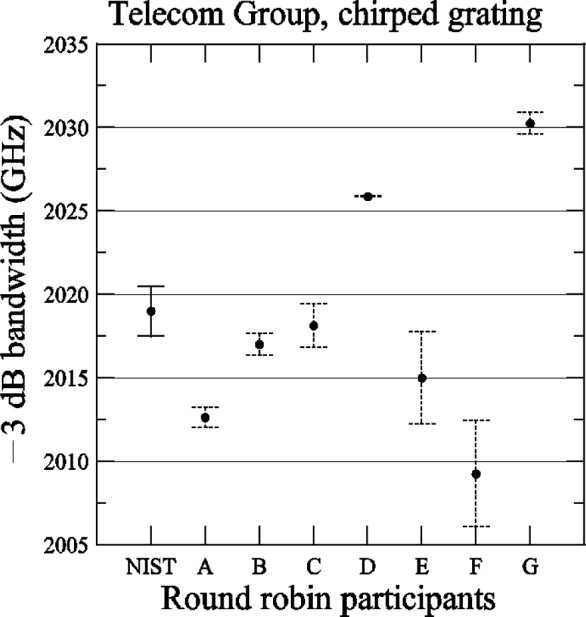
Bandwidth of the Telecom Group’s chirped grating. NIST uncertainties are expanded uncertainties, coverage factor (*k* = 2) and include all known components of uncertainty. Participants’ uncertainties are the fit uncertainty only (two standard deviation estimate).

Figure 14 shows the −3 dB center wavelength measurement for the chirped grating. The uncertainties shown for NIST represent the expanded uncertainty *U* = 6 pm (*k* = 2) of this measurement system. The uncertainties shown for the participants are due to the uncertainty of the fit only (two standard deviation estimate). The range of values is 165 pm with a standard deviation *s* of 57 pm. Figure 15 shows the −3 dB band width of the chirped grating. The uncertainties shown are similar to those in Fig. 14 converted to frequency. The range is 21 GHz with a standard deviation *s* of 7 GHz.

**Fig. 16:**
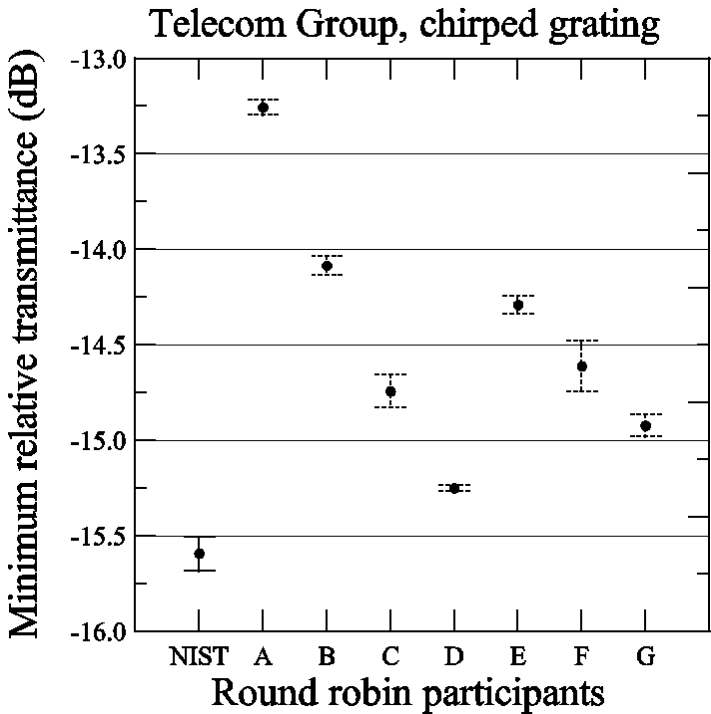
Minimum relative transmittance of the Telecom Group’s chirped grating. Uncertainties are only the uncertainty of the fit.

Figure 16 shows the minimum relative transmittance of the chirped grating determined from the data shown in Appendix C, Fig. 32. As stated earlier, the minimum transmittance measurement is sensitive to the spectral purity of the laser light source. NIST’s filtered laser shows the lowest minimum transmittance. The uncertainties shown (for NIST and the round robin participants) are only the uncertainty of the fit (two standard deviation estimate).

**Fig. 17:**
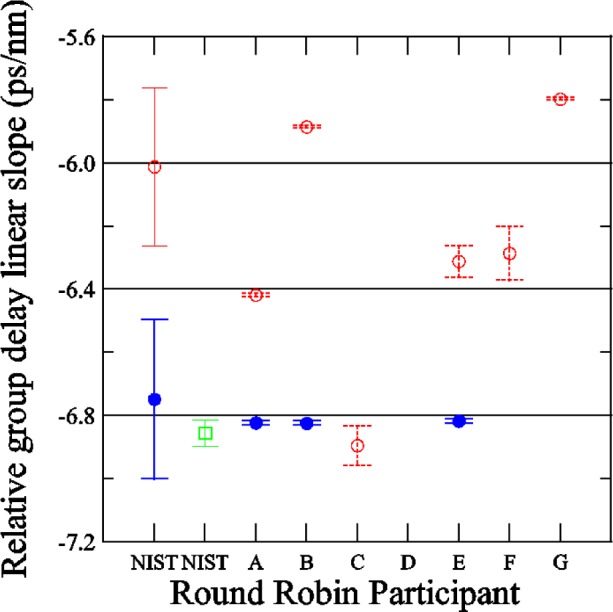
Linear slope of the *RGD* of the Telecom Group’s chirped grating. Data taken in only one direction on the grating are represented by ○ The mean of data taken in both directions on the grating are represented by ●. Data taken with the low-coherence system are represented by □ NIST uncertainties are expanded uncertainties, coverage factor (*k* = 2) and include all known components of uncertainty. Participants’ uncertainties are the fit uncertainty only (two standard deviation estimate).

Figure 17 shows the *RGD* linear slope determined from the data in Appendix C, Fig. 33. The linear slope was found using a least-squares-fit of a linear function to the −3 dB bandwidth of the data (see Appendix F). The uncertainties shown in Fig. 17 represent the repeatability (two standard deviation estimate) for NIST measurements using the phase-shift and low-coherence systems. The uncertainties for the participants represent only the fit uncertainty (two standard deviation estimate).

Figure 17 shows several values for the *RGD* linear slope due to a systematic bias that the phase-shift systems have. Because these systems are highly coherent they measure the *RGD* of the entire interferometer, not just the grating. Thus, the ≈10 m of fiber on each side of the FBG plus any fiber in the measurement system between the modulator and the detector will add some *RGD* to the measurement of the grating. To remove the bias from the surrounding fiber, the participant should make *RGD* measurements from both directions of the grating and, assuming the grating’s absolute *RGD* is is independent of direction, subtract the surrounding fiber *RGD*. NIST and participants A, B, and E provided data for both directions.

Shown in Fig. 17 are both the *RGD* linear slope for one direction, input on No. 1 (see Fig. 1), and the mean of both directions. Also included is the *RGD* measured with NIST’s low-coherence interferometer system. The low-coherence system has a better repeatability than the phase-shift system, possibly because the low-coherence system has faster data acquisition and does not require temperature-sensitive components such as the Mach-Zender modulator. The two NIST systems agree well, differing by about 0.1 ps/nm. The range for the value of the *RGD* linear slope, for one direction only, is 1.1 ps/nm with a standard deviation *s* of 0.4 ps/nm. The range for the mean of both directions and the low-coherence values of the *RGD* linear slope is 0.1 ps/nm with a standard deviation *s* of 0.04 ps/nm. The mean value is −6.81 ps/nm.

**Fig. 18:**
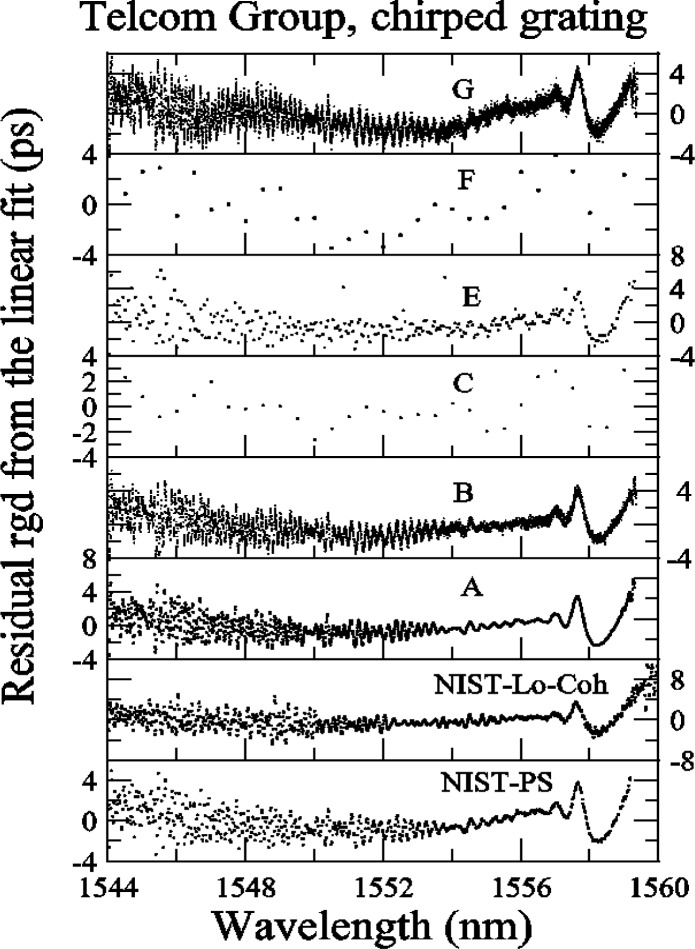
Residuals of the *RGD* linear fit for the Telecom Group’s chirped grating.

Figure 18 shows the residuals of the linear fit, across the −3 dB bandwidth, for each participant. To compare the agreement between NIST and participants A, B, E, and G, we calculated the difference from the mean residual at each wavelength and the standard deviation *s* from the mean residual. To compare the finer data sets with the coarser data sets, we compressed the finer data by determining a mean residual value using several data points over a small wavelength interval that matched the coarser data interval.

**Fig. 19:**
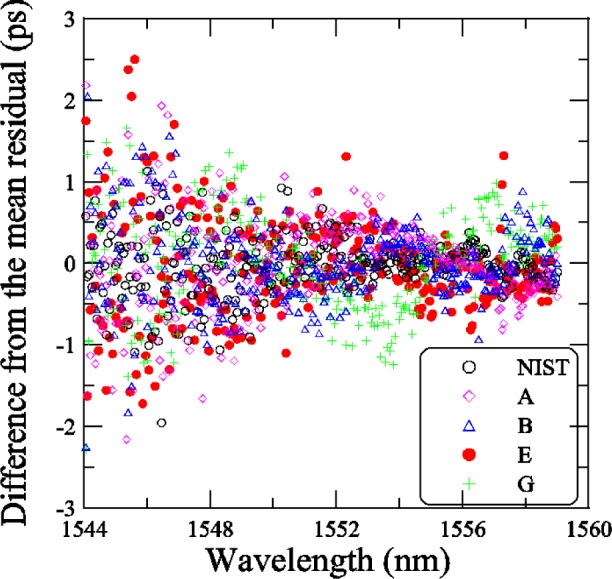
The difference from the mean residual for the *RGD* of the Telecom Group’s chirped grating.

**Fig. 20:**
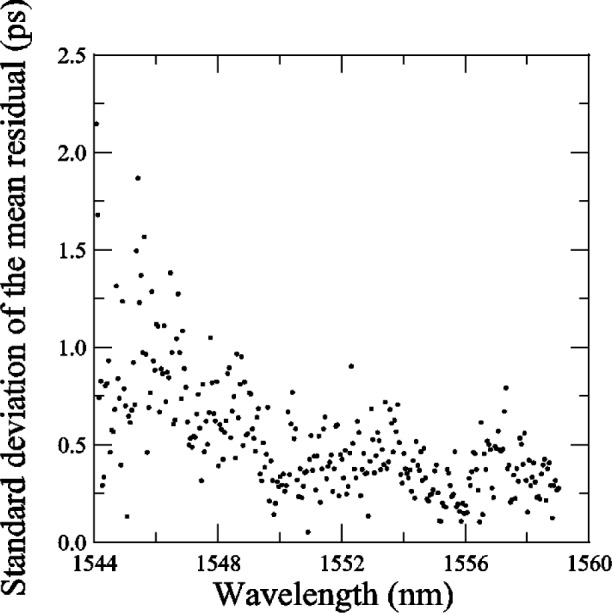
The standard deviation of the mean *RGD* residual of the Telecom Group’s chirped grating.

Figure 19 shows the difference from the mean residual and Fig. 20 the standard deviation *s* of the mean residual. The agreement at each wavelength interval is quite good, on average about 1 ps for the difference from the mean residual. The standard deviation *s* at most wavelengths is less than 1 ps. Thus, the various phase-shift systems record the same *RGD* ripple value to within 1 ps for *RGD* values ranging from about 40 to 150 ps. The agreement with the low-coherence system is still being improved. The current rms difference is 1.5 ps [2].

**Fig. 21:**
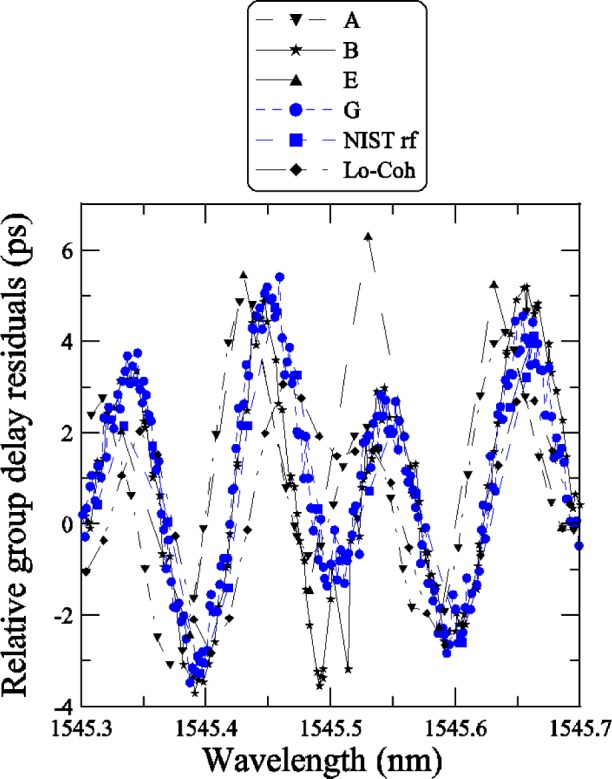
A portion of the *RGD* ripple from the chirped FBG as measured by various systems of the participants and NIST.

Figure 21 shows a portion of the residual *RGD* spectra taken with the NIST rf and low-coherence systems and with the systems of participants A, B, E, and G. For most cases the ripple measurements agree, but wavelength accuracy, measurement uncertainty, and rf sideband averaging can lead to differences of several picoseconds [5]. The difference between the low-coherence and rf phase shift systems is still being investigated, but no major differences have been observed [2]. The other round robin participant’s *RGD* data could not be used to compare the ripple because of coarse wavelength steps. Figure 20 illustrates the need for precision *RGD* ripple measurements, because over a 0.5 nm wavelength span the *RGD* changes rapidly, i.e., from +5 to −4 ps. Chirped gratings with larger *RGD* linear slopes will have larger *RGD* ripple amplitudes, increasing the need for more precision in *RGD* ripple measurements for WDM systems.

## 5. Sensors Group

### 5.1 Grating History

In this section a history of the −3 dB center wavelength of each of the sensor gratings is given.

**Fig. 22:**
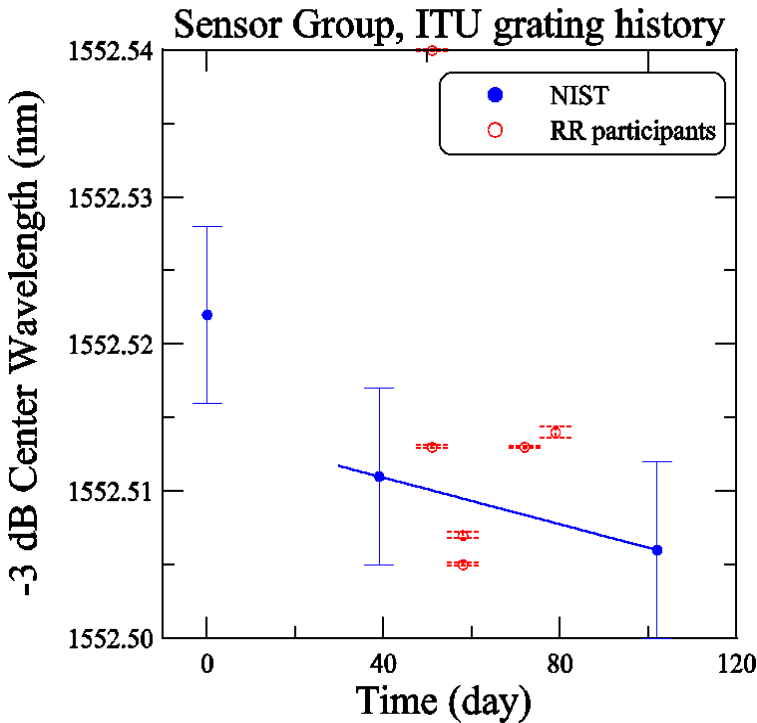
History of the Sensors Group’s ITU grating center wavelength. NIST uncertainties are expanded uncertainties, coverage factor (*k* = 2) and include all known components of uncertainty. Participants’ uncertainties are the fit uncertainty only (two standard deviation estimate).

Figure 22 shows the −3 dB center wavelength versus time for the Sensors group ITU grating. The NIST measurements are indicated by solid dots and the round robin participants by open circles. From the time of the construction of the round-robin box until the beginning of the round robin, the center wavelength changed by about 12 pm. After the round robin was completed the center wavelength showed an almost insignificant, change of about 5 pm, which is within the expanded uncertainty (coverage factor *k* = 2). The uncertainties on the NIST data represent the uncertainty of our measurement system, 6 pm (two standard deviation estimate). The uncertainties on the round-robin participants’ measurements represent only the fit uncertainty (two standard deviation estimate). The center wavelength is determined from the mean of the −3 dB wavelength values.

**Fig. 23:**
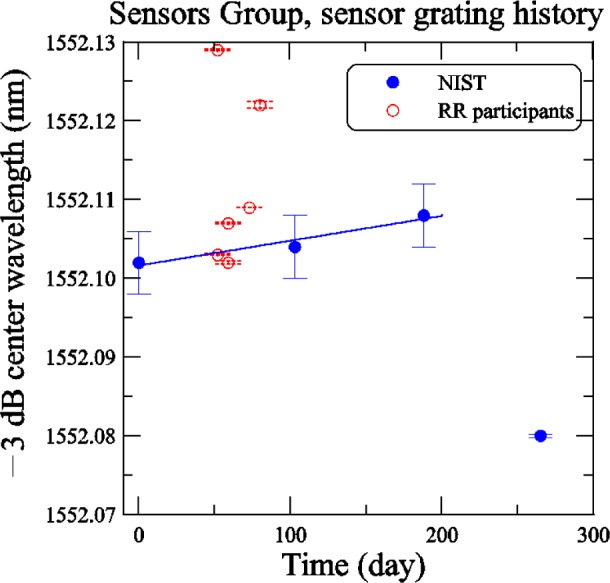
History of the Sensors Group’s sensor grating center wavelength. NIST uncertainties are expanded uncertainties, coverage factor (*k* = 2) and include all known components of uncertainty. Participants’ uncertainties are the fit uncertainty only (two standard deviation estimate).

Figure 23 shows the −3 dB center wavelength versus time for the Sensors Group sensor grating. The NIST measurements are indicated by solid dots and those of the round robin participants by open circles. From the construction of the grating until the end of the round robin, the center wavelength changed by about 2 pm, which is insignificant compared to the 4 pm expanded uncertainty (*k* = 2) shown in the figure. The grating showed further drift over 188 days of about 4 pm. The smaller uncertainty for this grating is due to the narrow bandwidth and shape of the reflectance profile. Because the center wavelength change was so small, no correction was added to the values of the participants.

These histories show that the gratings remained stable enough for the Sensors Group round robin to be useful.

### 5.2 Summary of Sensors Group Results

From the data received from each round-robin participant, the following parameters were determined: the center wavelengths and bandwidths were found at −3 dB from the relative reflectance of the ITU and sensor gratings. From the relative transmittance data the minimum relative transmittance was determined. Some of the participants in the Sensors Group gave two sets of data taken with different measurement systems. For the participants that gave two data sets, we labeled these with a numeral 0 or 1 following the letter used to designate the participant.

#### 5.2.1 ITU Grating

Some of the participants’ measurement systems did not have a large dynamic range and out-of-band features are missing (See Appendix D, Fig. 34). If we were to measure the isolation of this grating with these systems, we would obtain false values. However, because the Sensors Group was concerned primarily with wavelength accuracy, the isolation was not measured for these data. Also, from the relative transmittance data presented in Appendix D, Fig. 35 the dynamic range of a participant’s measurement system will affect the measurement of the minimum relative transmittance. The following figures show the center wavelength, bandwidth, and minimum transmittance.

**Fig. 24:**
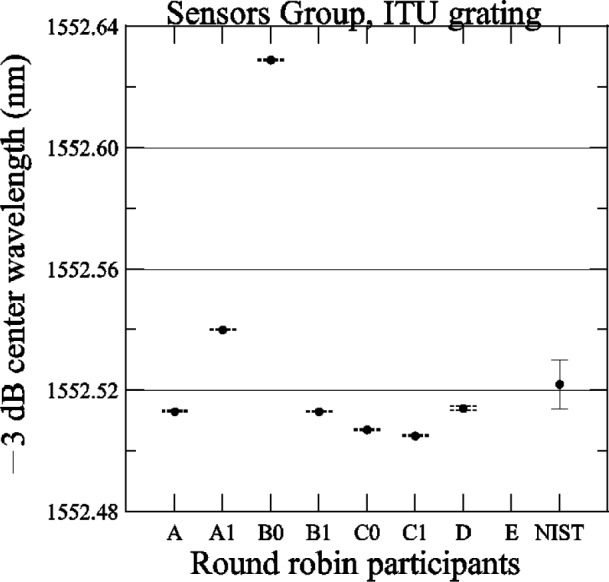
Center wavelength of the Sensors Group’s ITU grating. NIST uncertainties are expanded uncertainties, coverage factor (*k* = 2) and include all known components of uncertainty. Participants’ uncertainties are the fit uncertainty only (two standard deviation estimate).

Figure 24 shows the results of the center wavelength determined at −3 dB. The expanded uncertainty for the NIST measurement is *U* = 6 pm (*k* = 2). The uncertainties for the participants are just the fit uncertainty (two standard deviation estimate). The −3 dB center wavelength range of values is 124 pm, the standard deviation *s* is 42 pm, and the mean is 1552.530 nm. If the value of participant B0 is removed, the range of values is 35 pm, the standard deviation *s* is 12 pm, and the mean is 1552.516 nm. These mean values are only +4 pm and 10 pm (excluding B0) from the ITU channel 0 specified wavelength of 1552.526 nm. For most participants, this grating would pass the Telcordia specification, which requires that the specified and actual wavelength differ by less than 20 % of the bandwidth (80 pm) [4].

**Fig. 25:**
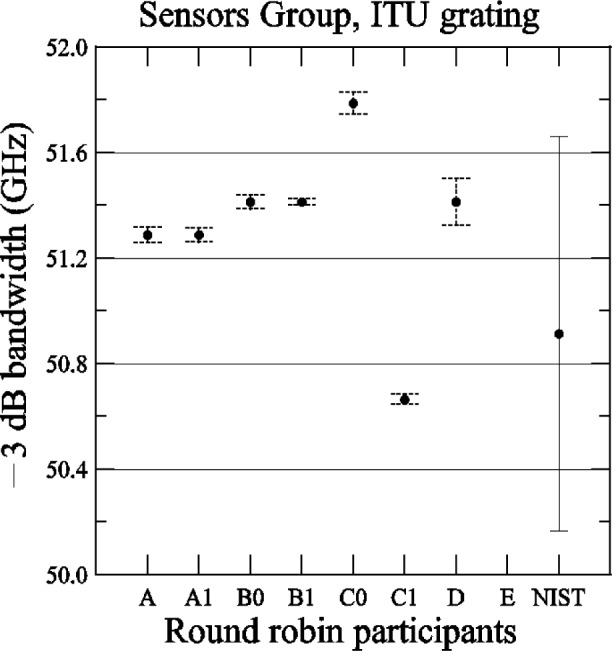
Bandwidth of the Sensors Group’s ITU grating. NIST uncertainties are expanded uncertainties, coverage factor (*k* = 2) and include all known components of uncertainty. Participants’ uncertainties are the fit uncertainty only (two standard deviation estimate).

Figure 25 shows the results of the bandwidth determined at −3 dB. The uncertainties are the same as discussed for Fig. 24 converted to GHz (the NIST uncertainty is 748 MHz, expanded uncertainty *k* = 2). For the −3 dB bandwidth the range of values is 1 GHz, the standard deviation *s* is 0.3 GHz, and the mean is 51.2 GHz.

**Fig. 26:**
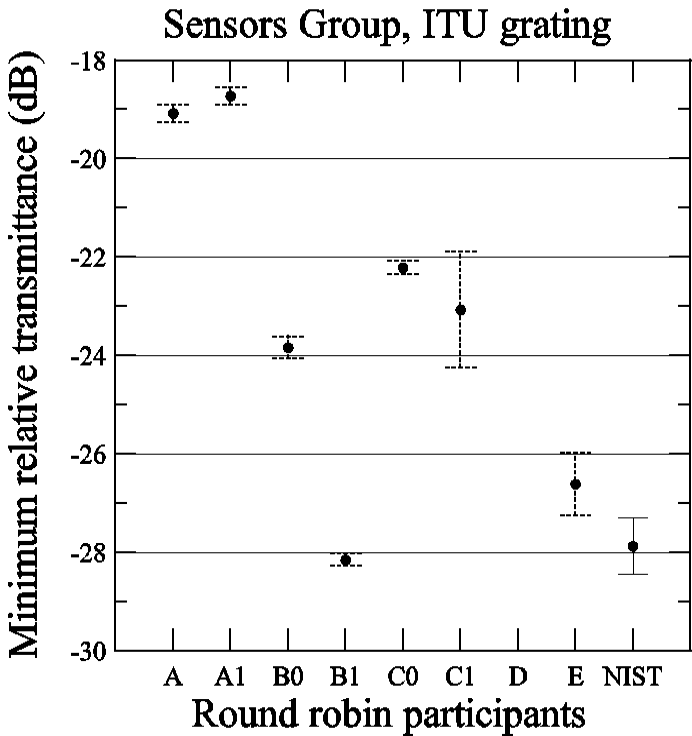
Minimum relative transmittance of the Sensors Group’s ITU grating. NIST uncertainties are expanded uncertainties, coverage factor (*k* = 2) and include all known components of uncertainty. Participants’ uncertainties are the fit uncertainty only (two standard deviation estimate).

Figure 26 shows the values for the minimum transmittance taken from the relative transmittance data shown in Appendix D, Fig. 35. The minimum transmittance measurement is sensitive to the spectral purity of the laser light source or filter bandwidth of the detector. For some participants, the minimum relative transmittance was as much as 9 dB lower than others. The uncertainties shown are only the uncertainty of the fit (two standard deviation estimate).

#### 5.2.2 Sensor Grating

In Appendix D, Figs. 36 and 37, the round robin data for the sensor grating shows that most participants measured with a fine enough wavelength interval, but with some participants the dynamic range of the system was low, rendering the measurement insensitive to detailed features. However, because this grating has a low relative reflectance, < 40 %, the minimum relative transmittance is not as sensitive to the dynamic range of the system or spectral purity of the source.

**Fig. 27:**
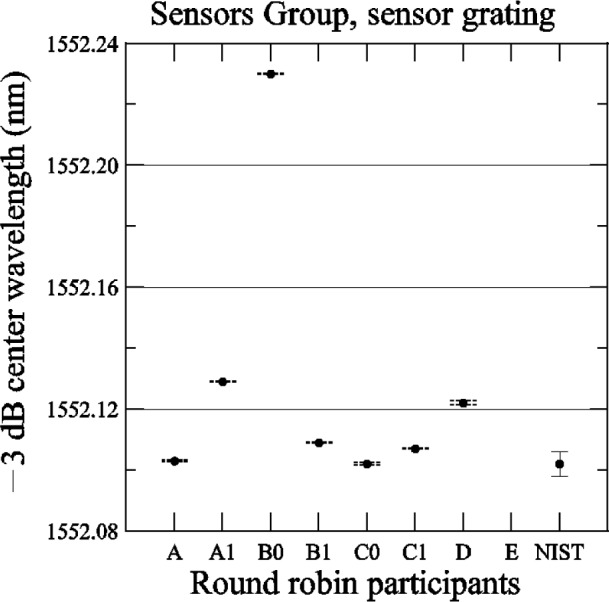
Center wavelength of the Sensors Group’s sensor grating. NIST uncertainties are expanded uncertainties, coverage factor (*k* = 2) and include all known components of uncertainty. Participants’ uncertainties are the fit uncertainty only (two standard deviation estimate).

Figure 27 shows the results of the center wavelength determined at −3 dB. The expanded uncertainty for the NIST measurement is *U* = 4 pm (*k* = 2). The uncertainties for the participants are just the fit uncertainty (two standard deviation estimate). The −3 dB center wavelength range of values is 128 pm, the standard deviation *s* is 43 pm, and the mean is 1552.125 nm. If the value of participant B0 is removed, the range of values is 27 pm, the standard deviation *s* is 11 pm, and the mean is 1552.111 nm.

If this were a typical sensor grating in a strain or temperature sensing application, the range of values for the center wavelength, 27 pm, converts to about a strain of 25 × 10^−6^ or about a 3 °C temperature variation. In some applications this variation would be acceptable and in others the variation would be too high.

**Fig. 28:**
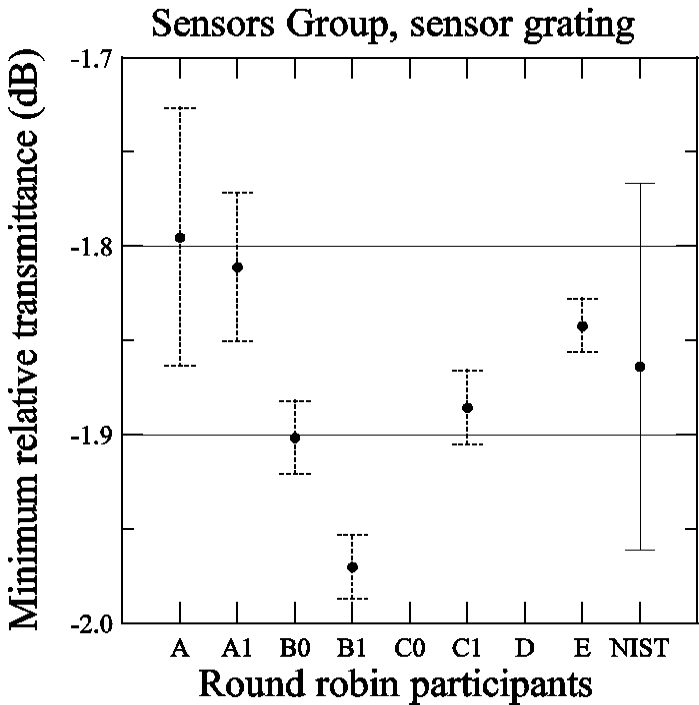
Minimum relative transmittance of the Sensors Group’s sensor grating. NIST uncertainties are expanded uncertainties, coverage factor (*k* = 2) and include all known components of uncertainty. Participants’ uncertainties are the fit uncertainty only (two standard deviation estimate).

**Fig. 29:**
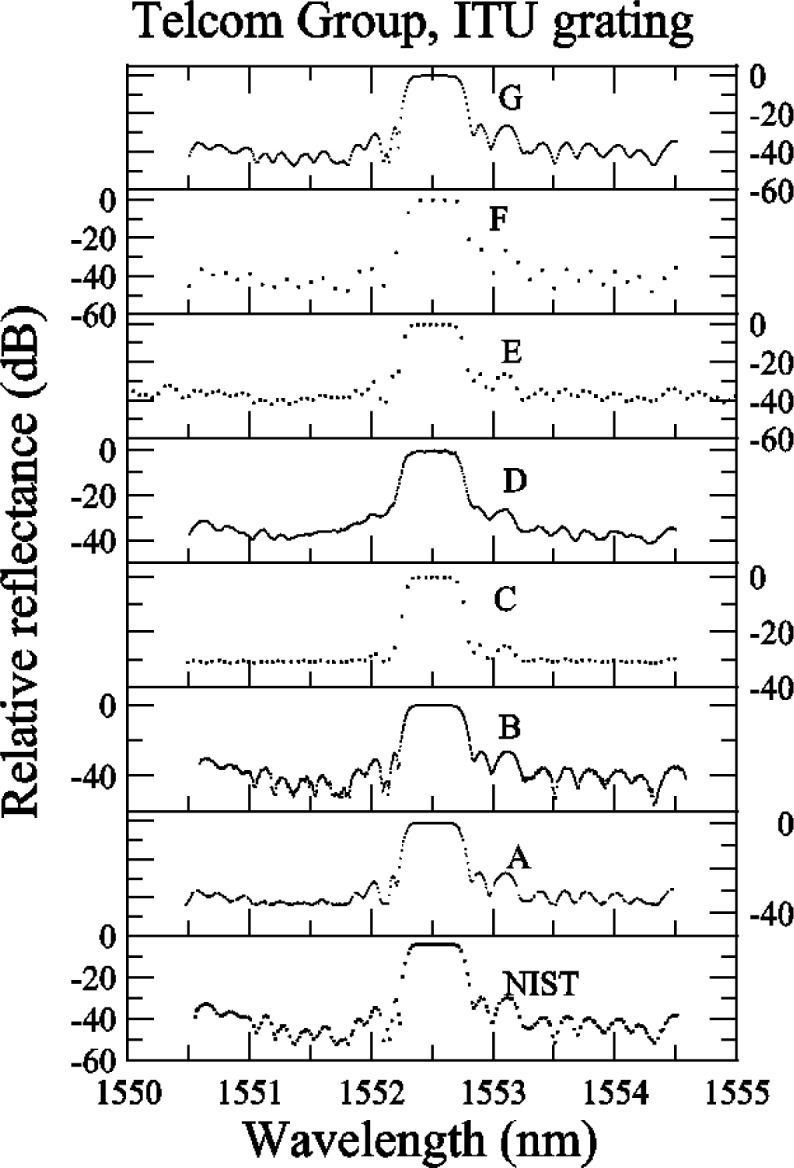
Telecom Group’s relative reflectance data for the ITU grating.

**Fig. 30:**
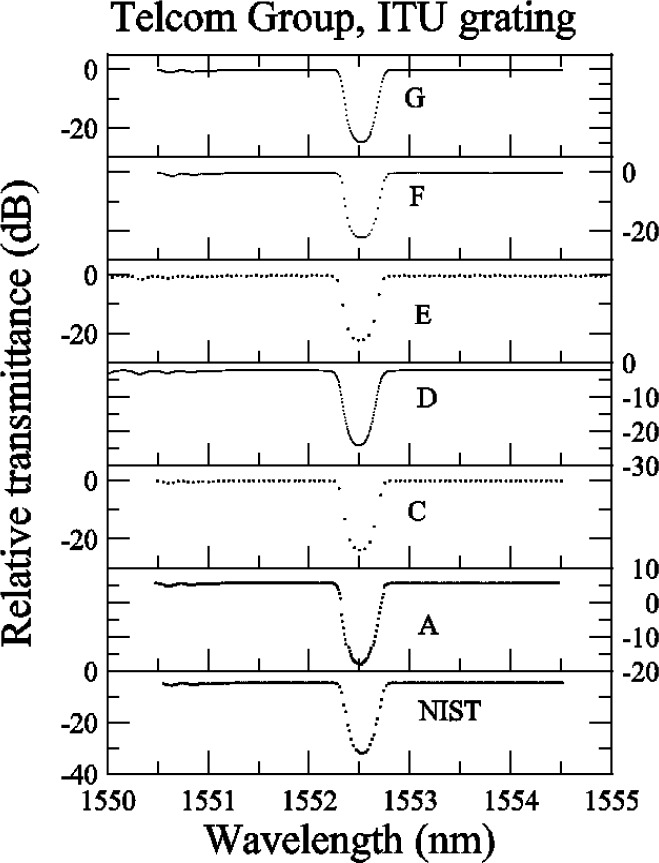
Telecom Group’s relative transmittance data for the ITU grating.

**Fig. 31:**
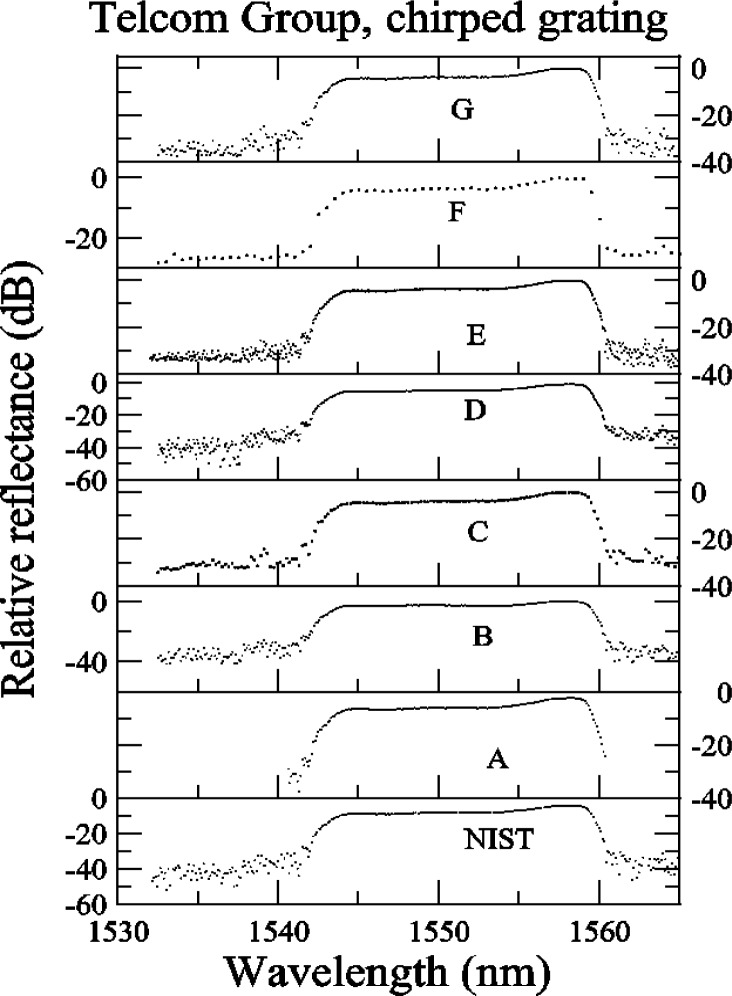
Telecom Group’s relative reflectance data for the chirped grating.

**Fig. 32:**
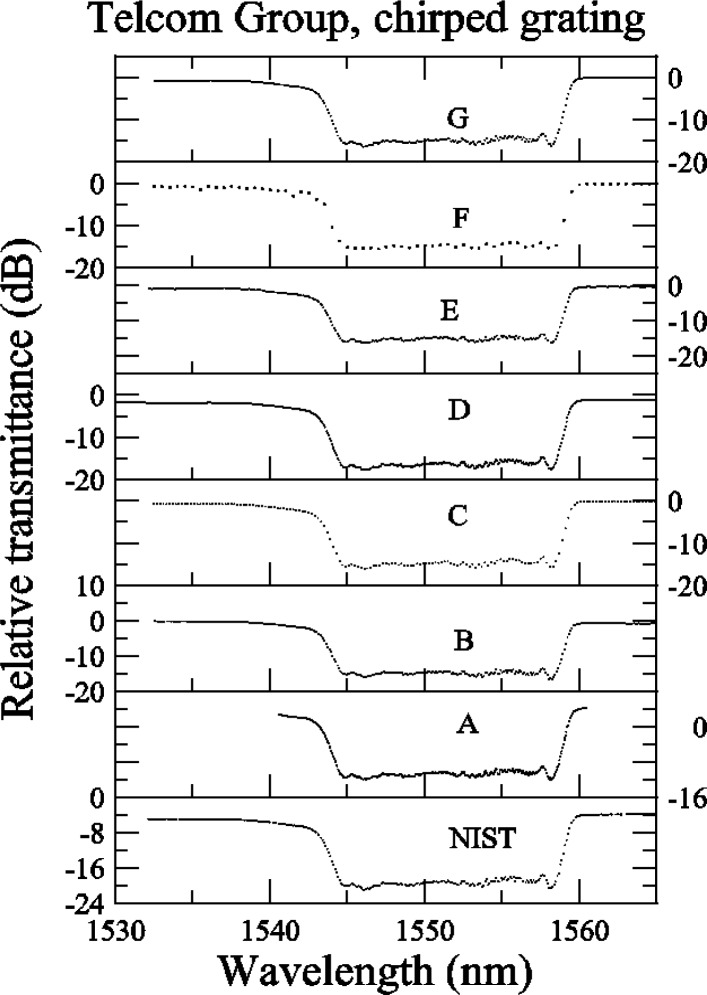
Telecom Group’s relative transmittance data for the chirped grating.

**Fig. 33:**
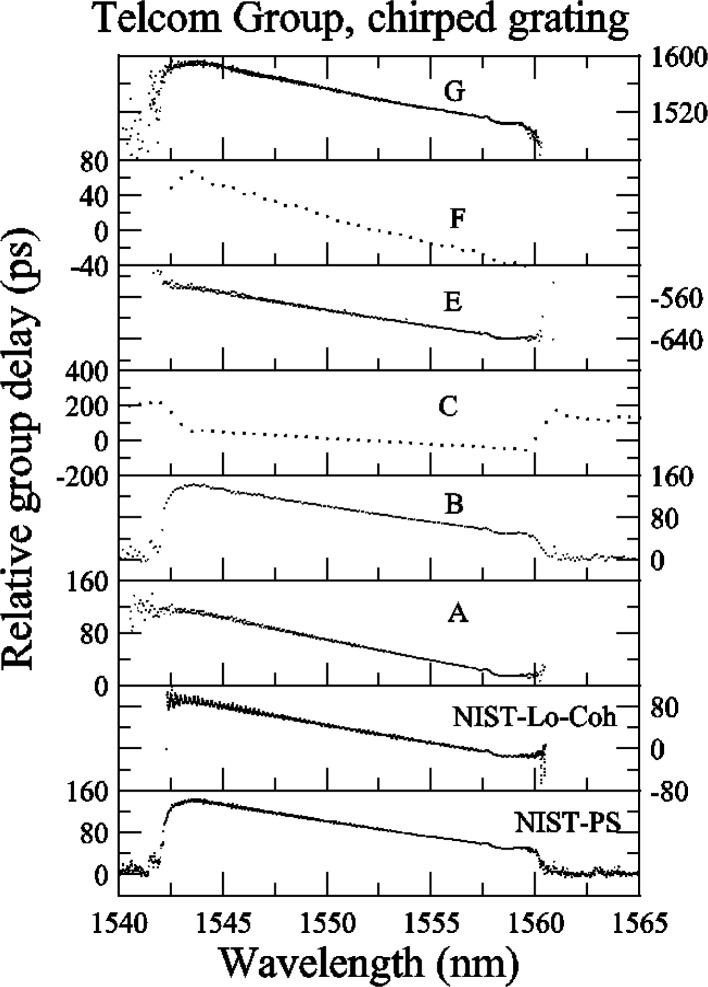
Telecom Group’s *RGD* data for the chirped grating.

**Fig. 34:**
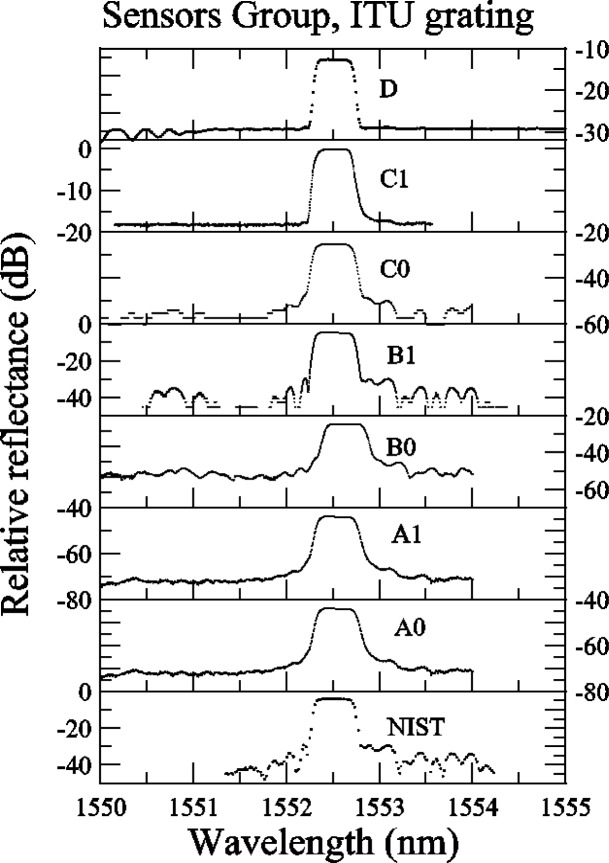
Sensors Group’s relative reflectance data on the ITU grating.

**Fig. 35:**
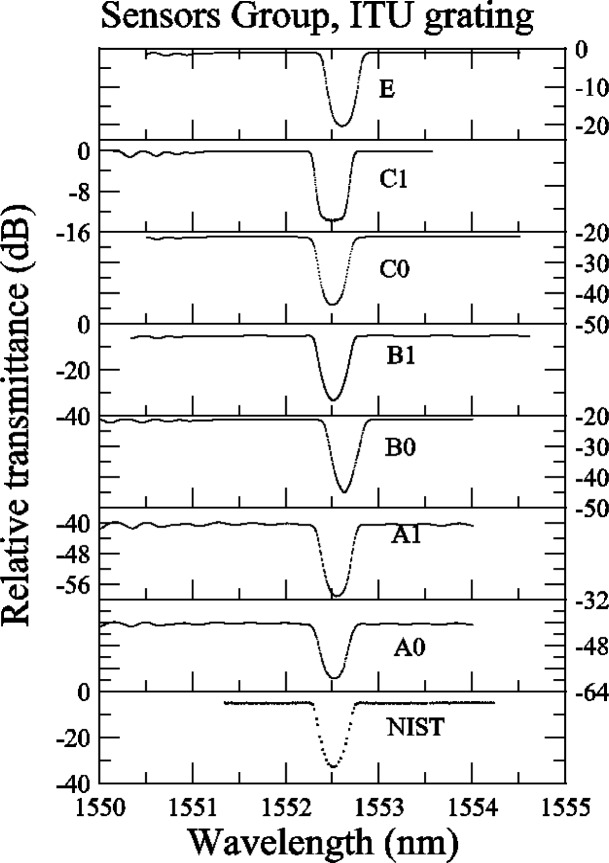
Sensors Group’s relative transmittance data on the ITU grating.

**Fig. 36:**
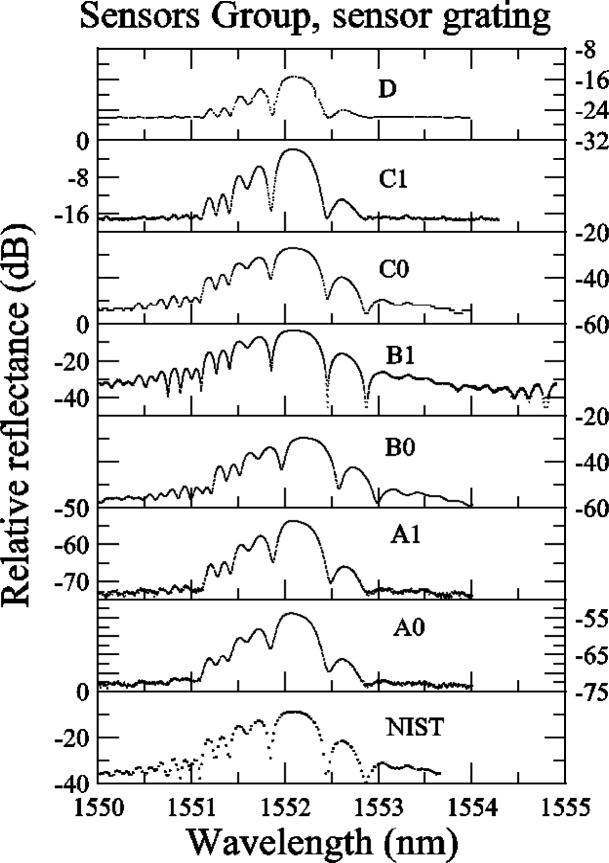
Sensors Group’s relative reflectance data for the sensor grating.

**Fig. 37:**
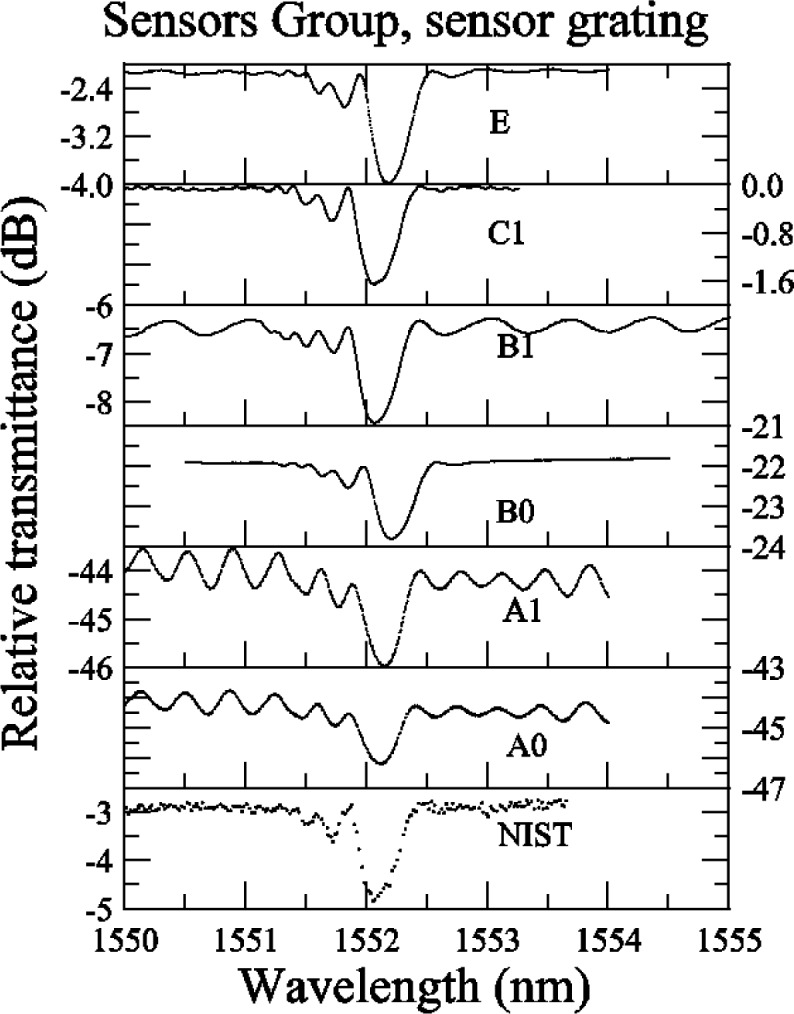
Sensors Group’s relative transmittance from the sensor grating.

Figure 28 shows the values for the minimum transmittance taken from the relative transmittance data shown in Appendix D, Fig. 37. The uncertainties shown for the participants are only the uncertainty of the fit (two standard deviation estimate).

## 6. Conclusions

Metrology for WDM components, such as FBGs, must improve to meet the demands of current and future WDM networks. From the round-robin results we can draw the following conclusions. The state of FBG metrology appears inadequate for measurements of isolation, minimum relative transmittance, bandwidth, and *RGD* linear slope.

The source spectral purity is critical; ASE from diode sources must be substantially reduced, and more care is needed among the two groups concerning ASE. The spectral purity or detector filter bandwidth is important when measuring transmittance. The bandwidth of the optical spectrum analyzer (OSA) must be carefully considered when this instrument is used.

Wavelength uncertainty of <1 pm and step sizes <10 pm are necessary for bandwidth and *RGD* ripple measurements. Removal of fluctuations in the source spectral power is necessary to measure bandwidth. The criteria for determining the center wavelength and bandwidth appear to be important for ITU gratings. The use of the average of the −3 dB points may be standard practice but is not as practical for the system designer as using the −0.5 dB points. If −0.5 dB is accepted by industry as the value used to determine the center wavelength and bandwidth, many gratings being produced and in use will not pass the bandwidth specification. The center wavelength value did not change significantly for either the −3 dB or −0.5 dB criterion.

Comparing the two groups for the measurement of the center wavelength of the ITU gratings, we note that the sensors group had a 35 pm range and *s* = 12 pm, while the telecom group had a 28 pm range and *s* = 8 pm. The telecom group primarily relied on wavelength meters to set the wavelength scale and the sensors group relied on OSAs. Surprisingly, this comparison would indicate that there is little difference between the two systems when measuring the ITU grating.

Sensor applications need better absolute calibration on the wavelength scale. The spread in values for the center wavelength of the sensor grating will not meet the needs of long-term structural monitoring.

The measured *RGD* linear slope of the chirped grating had a range of 16 % when only one direction of the grating was measured. When using rf phase-shift systems, the best way to remove the system bias is to take the mean of both directions on the grating. When both directions were used the *RGD* linear slope range was 1.5 %.

If the same relative range of *RGD* linear slope (16 %) were applied to a 100 km dispersion compensating grating, the effects on a telecommunication link would be dramatic; the uncertainty in the dispersion compensating grating would be around 100 ps/nm. The actual industry measurement range for *RGD* on a 100 km dispersion compensating grating is probably less than this. A round robin for this type of grating would provide a useful check.

*RGD* ripple values have ±2 ps differences among participants. Stabilizing rf phase-shift measurement systems and working at lower rf frequencies so the wavelength resolution is <10 pm is necessary for *RGD* ripple measurements. A standard way of discussing or presenting the *RGD* ripple is needed. Also, *RGD* resolution <1 ps will be required for most WDM components as data rates increase. *RGD* measurements with the low-coherence system compare well with phase-shift systems and may be preferred for rapid evaluation of components.

This round robin with FBGs showed that with proper packaging and monitoring, reasonable results for a comparison could be achieved. The gratings used in this round robin, when handled well by the shipper, showed little change in central wavelength. Those gratings that endured a large shock/acceleration showed moderate drift but, with interval monitoring at NIST, corrections could be made.

## Figures and Tables

**Fig. 38:**
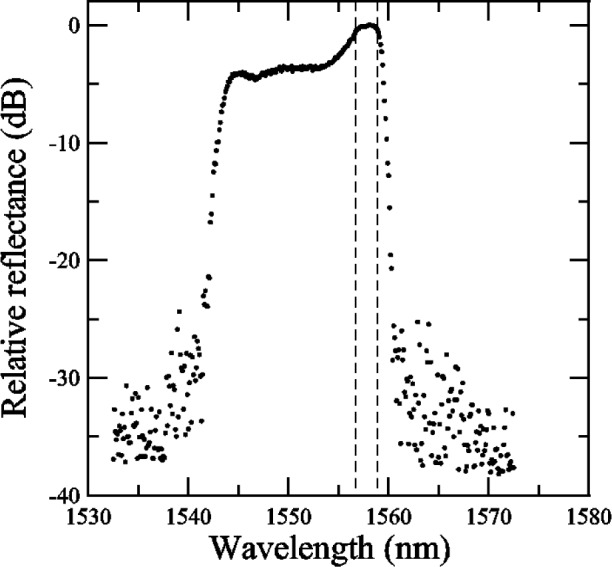
Lower and upper −0.5 dB wavelengths shown as vertical dotted lines.

**Fig. 39:**
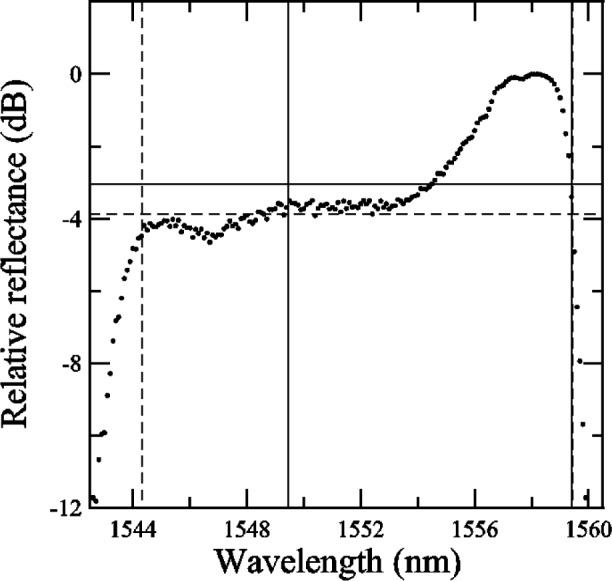
The −0.5 dB wavelengths based on the *shorth* (dashed line) and the mean (solid line) (see Appendix G).

## Footnotes

1 Certain commercial equipment, instruments, or materials are identified in this paper to foster understanding. Such identification does not imply recommendation or endorsement by the National Institute of Standards and Technology, nor does it imply that the materials or equipment identified are necessarily the best available for the purpose.

## 7. Appendix A. Telecom Group, Bragg Grating Round Robin Procedure

1. Once you receive the package, open it up and splice the gratings into your measurement system. The Chirped grating has fiber numbers 1 and 2, the ITU grating has fiber numbers 3 and 4. For all measurements please record the input fiber number. If you make only one dispersion measurement of each grating, launch into fiber No. 1 for the chirped and fiber No. 4 for the ITU.
2. Apply power to the unit, enable the thermoelectric cooler (TEC) by pressing the green button, and allow about a 30 min warmup period. The thermometer should read 25.0 °C. (If not call me at 303-497-5599.)
3. Begin your measurements:
  **Table:**
  • ITU grating	• 4 nm wide, high resolution (<0.1 nm steps), scan over the reflectance/transmittance window; suggested range is 1550.5 nm to 1554.5 nm.
  	• 40 nm wide, low resolution (0.1 nm to 0.2 nm steps), reflectance/transmittance scan; suggested range 1532.5 nm to 1572.5 nm.
  Chirped grating	• 40 nm wide, low resolution, reflectance/transmittance scan, suggested range 1532.5 nm to 1572.5 nm.
  	• Relative dispersion measurement over a 25 nm wide scan.
4. [4] Send your results by email, preferably as an attached file (my address is arose@boulder.nist.gov). The data for each scan should be in ASCII format, two columns [first column wavelength (nm), second column voltage or optical power (dB) or relative dispersion (ps), optional third column temperature (° C)].
  In your email message describe your measurement methods, such as stepped tunable laser and spectrum analyzer, etc. If the temperature is not listed in the third column of your data set, mention it in the text of your message.
5. [5] Lock the box up and send it on to the next participant by Friday or the date indicated on the schedule. We have included an envelope with a Federal Express shipping label for you to affix to the outside of the box. Please make sure you are sending it to the next participant. Please put the envelope back in the shipping box for the next participant to use. Thanks to all.

## 8. Appendix B. Sensors Group, Bragg Grating Round Robin Procedure

1. Once you receive the package, open it up and splice the gratings into your measurement system. The Sensor grating has fiber numbers 1 and 2, the ITU grating has fiber numbers 3 and 4. For all measurements please record the input fiber number.
2. Apply power to the unit, enable the thermoelectric cooler (TEC) by pressing the green button, and allow about a 30 min warmup period. The thermometer should read 25.0 °C. (If not call me at 303-497-5599.)
3. Begin your measurements:
  **Table:**
  ITU grating	• 4 nm wide, high resolution (<0.1 nm steps), scan over the reflectance/transmittance window; suggested range 1550.5 nm to 1554.5 nm.
  	• 40 nm wide, low resolution (0.1 nm to 0.2 nm steps), reflectance/transmittance scan; suggested range 1532.5 nm to 1572.5 nm.
  Sensor grating	• 4 nm wide, high resolution, reflectance/transmittance scan, suggested range 1550 nm to 1554 nm.
  	• 40 nm wide, low resolution (0.1 nm to 0.2 nm steps), reflectance/transmittance scan; suggested range 1532 nm to 1572 nm.
4. Send your results by email, preferably as an attached file (my address is arose@boulder.nist.gov). The data for each scan should be in ASCII format, two columns [first column wavelength (nm), second column voltage or optical power (dB), optional third column temperature (° C)].
  In your email message describe your measurement methods, such as stepped tunable laser and spectrum analyzer, etc. If the temperature is not listed in the third column of your data set, mention it in the text of your message.
  In your email message, please give the center wavelength, reflectance, and bandwidth values for the ITU and Sensor gratings.
5. Lock the box up and send it on to the next participant by Friday or the date indicated on the schedule. We have included an envelope with a Federal Express shipping label for you to affix to the outside of the box. Please make sure you are sending it to the next participant. Please put the envelope back in the shipping box for the next participant to use. Thanks to all.

## 11. Appendix E. NIST Uncertainty

The uncertainty of various measurements will be discussed in this section. Because only raw data were given to NIST, only a fit uncertainty can be attributed to various parameters determined from a participant’s data. However, NIST has determined the complete uncertainty of the various measurement systems it uses and those uncertainties are included in the uncertainties associated with parameters determined from NIST data.

The NIST system used to measure the spectral properties of a grating, shown in Fig. 2, has the following attributes. The laser has a stability of about 80 MHz over a 10 s period, with a line width less than 300 kHz, and a −3 dB tuning range of 1525 nm to 1585 nm. The tunable fiber Fabry-Perot filter has a −3 dB bandwidth of about 1.5 GHz. The tunable filter attenuates the amplified spontaneous emission from the diode laser as shown in Fig. 3. The peak transmittance of the filter is dithered in wavelength across the peak output of the laser, which modulates the laser’s transmitted intensity. This modulated laser intensity used with a lock-in amplifier allows a dynamic range of about 68 dB. The wavelength meter in this system has a wavelength resolution of ±0.1 pm and an expanded uncertainty of 0.2 pm (*k* = 2). The wavelength meter uncertainty is periodically checked by comparison with a rubidium absorption cell.

The NIST repeatability of determining the center wavelength and bandwidth of the round robin ITU grating, including the wavelength meter and fit uncertainty, was *U* = 6 pm (*k* = 2). We find that for a narrow-band sensor-type grating the repeatability is ±4 pm (two standard deviation estimate). Included in the fit uncertainty are the effects of the coupler transmittance versus wavelength on the reflectance of the grating. After further investigation, we found the method we used to step the laser wavelength caused the laser wavelength to drift during a measurement and the laser did not step evenly in wavelength. A new method was used and the repeatability of our measurements is now limited by the wavelength meter’s uncertainty. The gratings are in an athermal package that has a 0.5 pm/°C temperature dependence, and the cooler has a temperature stability of about 0.01 °C, which should provide an estimated wavelength stability of 0.01 pm.

The uncertainty of the absolute transmittance data is dominated by the uncertainty of the linearity and responsivity of the detectors used, which is about 1 % (two standard deviation estimate). We measured the linearity of the detectors over a 40 dB range of output voltages and found the linearity to vary less than ±0.1 % (two standard deviation estimate). We measured minimum relative transmittance to a level of less than −60 dB. Assuming a 1 % uncertainty in the linearity of the detector-lock-in-amplifier system, we have an uncertainty at −60 dB of about 2.5 dB (two standard deviation estimate). The wideband fiber couplers used have a small wavelength dependence. Because a reference measurement is taken from the unused port of the 2 × 2 coupler, much of the laser’s, and detector’s wavelength dependence can be removed from the transmittance and reflectance signals. The uncertainty of the reflectance is dominated by the uncertainty of the coupling ratios and the detector’s response. In this report only relative values are used because of differences in measurements systems. The coupler splitting ratio has a weak wavelength dependence of about 0.01 dB/nm over the 1540 nm to 1560 nm range.

The NIST system used to measure the *RGD* of the gratings, shown in Fig. 5, has an uncertainty of about 0.3 ps (two standard deviation estimate). However, for long-term measurements, the system can have a phase drift due to temperature changes of the modulator. Care must be taken to hold the temperature constant during a measurement or an additional bias is added to the *RGD* values. With the 1.92 GHz modulation used, the wavelength uncertainty is about 30 pm (two standard deviaton estimate).

A full uncertainty analysis of the white-light interferometer shown in Fig. 6 is still in progress.

## 12. Appendix F. Determination of Wavelength Increments

This Appendix describes the procedures we used to determine the appropriate data spacing for a transmission/reflectance response curve.

Let *y*_1_, *y*_2_,… *y*_n_ (in dB) be the measured response in the “top flat” area of the transmission/reflectance curve, then the −*x* dB transmission/reflectance response *y*_−_*_x_* is obtained as

$$y_{-x}=\max(y_{1},y_{2},\ldots y_{n})-x.$$ (1)

If there are no outlying data points, max(*y*_1_, *y*_2_,… *y_n_*) is the estimate of the “plateau” level of the curve. For smooth data sets, where the source wavelength dependence has been removed and noise is low, this assumption will hold. We can determine the proper sample size, hence the proper wavelength increment, based on the desired precision of this plateau estimate. If we assume the *y_i_* are independent and equally probable to lie anywhere within the interval *a* and *b* (i.e., the maximum possible measurement error is *b − a*), then it can be shown [6] that the standard deviation (*s*) of *y*_−_*_x_* is given by

$$s(y_{-x})=\sqrt{\frac{n}{\left(n+2\right){\left(n+1\right)}^{2}}}\left(b-a\right)\approx \frac{b-a}{n+2}.$$ (2)

We can then equate this standard deviation to a threshold value to obtain the sample size required. For example, if we want to have an estimate of −*x* dB transmission/reflectance response with a standard deviation less than one-tenth of the maximum possible error (in the top flat portion), we need to have at least eight measurements in that area.

Once we have a “good” estimate of the −*x* dB transmission/reflectance response, the lower and upper −*x* dB wavelengths can be calculated. We consider only the lower −*x* dB wavelength here. Let *y*^−^ and *y*^+^ be the first two consecutive measured responses such that *y*^−^ ≤ *y*_−_*_x_* ≤ *y*^+^. The corresponding wavelengths for *y*^−^ and *y*^+^ are λ_1_ and λ_1_ + *h* (*h* > 0), respectively. The lower −*x* dB wavelength based on linear interpolation is given by

$$\lambda_{L}=\lambda_{1}+\frac{y_{-x}-y^{-}}{y^{+}-y^{-}}h.$$ (3)

The maximum error of λ_L_ can be estimated by [7].

$$\Delta \lambda_{L}\approx \frac{\Delta y}{{dy/d\lambda }_{L}},$$ (4)

where Δ*y* is the maximum possible error in the transmission/reflectance measurements. An approximate value for d*y*/dλ_L_ is (*y*^+^ − *y*^−^)/*h*, or

$$\Delta \lambda_{L}\approx \frac{\Delta y}{y^{+}-y^{-}}h.$$ (5)

The bandwidth (λ^+^_−_*_x_* – λ^−^_−_*_x_*) determined from the transmittance/reflectance response will be effected by *h*, Δ*y*, and *y*^+^ − *y*^−^ as seen in Eq. (5). For a desired maximum error ϵ in Δλ_L_ (and the bandwidth), a wavelength step size can be found according to

$$h\leq \frac{\varepsilon (y^{+}-y^{-})}{\Delta y}.$$ (6)

The result in Eq. (6) indicates that when the response curve is slowly varying in regions where *y*_−_*_x_* is located (*y*^+^ − *y*^−^ is small), or Δ*y* is large, we need a smaller increment. When the response curve is slowly varying around *y*_−_*_x_* (*y^+^* − *y*^−^ is small), then according to Eq. (6)*h* will need to be smaller than if the response curve had a larger difference between *y*^+^ − *y*^−^. Also, if Δ*y* is large around *y*_−_*_x_*, then *h* will need to be smaller.

## 13. Appendix G. Robust Estimation of the −*x* dB Wavelengths

This Appendix describes the robust statistical method we used to determine the lower and upper −*x* dB wavelengths.

When there are outlying measurements, the lower and upper −*x* dB wavelengths based on the −*x* dB transmission/reflectance response *y*_−_*_x_* calculated as

$$y_{-x}=\max(y_{i},i=1,2,\ldots )-x$$ (7)

may be misleading. For example, the dotted vertical lines in Fig. 38 are the lower and upper −0.5 dB wavelengths. Obviously, the results reflect only the presence of the hump at the right side. Thus, we need a robust estimate of *y*_−_*_x_* representing the plateau level of the transmission/reflectance curve.

Let *y*_1_, *y*_2_,… *y_n_* be the measured responses in the upper region of the transmission/reflectance curve. This can be accomplished by accepting only the responses that are greater than a cutoff value. For the example in Fig. 38, we could use a cutoff value, say, −6 dB. A particular cutoff value is not critical; any reasonable value will yield almost identical results because of the robustness of the procedure.

One might use the mean $\bar{y}=\sum_{i=1}^{n}y_{i}/n$ to estimate the plateau level of the curve. However, the mean is sensitive to outliers. We propose two alternatives. The first is the median of *y_i_*. The second is a statistic, called *shorth*, which is similar to the median (in robustness) but has a convenient geometrical interpretation. The *shorth* of *y_i_*, *i* = 1, 2,… n, is the midpoint of the shortest interval that includes half of *y_i_*. This is done by finding the smallest of the values $y_{k}^{*}-y_{1}^{*}$, $y_{k+1}^{*}-y_{2}^{*}$,…, $y_{n}^{*}-y_{n-k+1}^{*}$, where *k* = [*n*/2] + 1, [*p*] is the integer part of *p*, and $y_{1}^{*}\leq y_{2}^{*}\leq \ldots \leq y_{n}^{*}$ are the ordered measurements of *y_i_*. Then, the *shorth* simply equals the midpoint of this shortest interval. For example, let the ordered measurements of *y_i_*, *i* = 1, 2,…, 100, be

134781415161727100.

Then *k* = [11/2] + 1 = 6 and the six intervals that include half of the measurements are

(1,14),(3,15),(4,16),(7,17),(8,27),(14,100).

The shortest interval is (7, 17) and the *shorth* = (17 + 7)/2 = 12. Note that the median of the above eleven measurements is 14, while the mean is 19.3 (skewed by a single measurement).

If we fit a horizontal line to *y_i_*, *i* = 1, 2,…, *n*, the mean of *y_i_* is the line that minimizes the *sum* of the squared residuals (differences between the predicted and measured *y_i_*). The *shorth* of *y_i_* is the line that minimizes the *median* of the squared residuals. The median is not affected by the values of the outlying residuals and will not change unless more than half the residuals represent bad or spurious measurements. In summary, the *shorth* is a robust estimate of the plateau level of the transmission/reflectance curve.

Figure 39 displays the estimated plateau of the transmission/reflectance curve based on the mean (solid horizontal line) and the *shorth* (dashed horizontal line) of *y_i_*. It also shows the −0.5 dB wavelengths based on the *shorth* (dashed vertical lines) and the mean (solid vertical lines).
